# Elucidation of Antimicrobials and Biofilm Inhibitors Derived from a Polyacetylene Core

**DOI:** 10.3390/molecules29245945

**Published:** 2024-12-17

**Authors:** Tyler L. Skeen, Rebekah L. Gresham, Katherine A. Agamaite, Olivia M. Molz, Isabelle F. Westlake, Sage M. Kregenow, Al K. Romero, Brian M. Flood, Lauren E. Mazur, Robert J. Hinkle, Douglas D. Young

**Affiliations:** Department of Chemistry, William & Mary, Williamsburg, VA 23185, USA; tlskeen@wm.edu (T.L.S.); rlgresham@wm.edu (R.L.G.); kagamaite@wm.edu (K.A.A.); ommolz@wm.edu (O.M.M.); ifwestlake@wm.edu (I.F.W.); smkregenow@wm.edu (S.M.K.); bmflood@wm.edu (B.M.F.); lemazur@wm.edu (L.E.M.)

**Keywords:** antibiotics, alkynes, biofilms, structure–activity relationships, Glaser–Hay reaction

## Abstract

The development of new antibiotics with unique mechanisms of action is paramount to combating the growing threat of antibiotic resistance. Recently, based on inspiration from natural products, an asymmetrical polyacetylene core structure was examined for its bioactivity and found to have differential specificity for different bacterial species based on the substituents around the conjugated alkyne. This research further probes the structural requirements for bioactivity through a systematic synthesis and investigation of new compounds with variable carbon chain length, alkynyl subunits, and alcohol substitution. Furthermore, the research examines the activity of the new compounds towards the inhibition of biofilm formation. Overall, several key new polyyne compounds have been identified in both decreasing bacterial viability and in disrupting pre-formed biofilms. These properties are key in the fight against bacterial infections and will be helpful in the further development of new antibiotic agents.

## 1. Introduction

Antibiotic resistance is a growing problem [1] with significant implications for human health. A United States CDC report suggests that antibiotic-resistant bacterial infections account for at least 35,000 deaths annually, and at least 18 different bacterial and fungal strains demonstrate some form of antibiotic resistance [2]. These strains cause 2.8 million infections every year in the U.S. alone, taking a significant social and economic toll. These numbers suggest that bacterially related infections will exponentially rise, rivaling cancer for global death rates by 2050. These dire circumstances necessitate the discovery of novel antibiotics with unique mechanisms of actions.

In addition to growing antibiotic resistance in bacteria, there is the looming threat of bacterial biofilm growth. Bacterial biofilms are a complex community of cells that have adhered to a surface by a self-produced polymer matrix composed of mainly polysaccharides, extracellular DNA, and secreted proteins [3]. Once formed, the matrix of sugars and proteins forms a protective layer conferring high resistance to traditional antibiotics [4]. One of the major implications of bacterial biofilm growth arises in the health sector. Bacterial biofilms have been observed on medical implants and ultimately lead to chronic infections. This is illustrated by the fact that infections connected to microorganism biofilms account for 80% of all chronic responses of organisms during hospitalization [5]. In addition to the simple ability to kill infectious bacteria and prevent biofilm growth, a significant need arises for therapeutics that can disrupt these biofilms after they have already formed.

One of the main methods of developing new biologically active antimicrobials is through exploring derivatives of natural products [6]. Among observed biologically active natural products, over 2500 contain at least two consecutive, conjugated alkynes (polyynes) [7,8,9,10,11,12,13], and new analogues continue to be discovered [14,15]. These polyynes exhibit a wide range of activities ranging from allelopathic [16] to butyrylcholinesterase (BChE) inhibitors [17] to antifungal and antimicrobial [18,19,20] and even potential anti-cancer drugs [21,22,23,24,25]. Based on these observations and a recently developed solid-supported Glaser–Hay synthetic methodology to rapidly generate polyynes asymmetrically, our previous research involved generating libraries of polyynes based on alkynes that also contain a primary alcohol moiety [26].

**Figure 1:**
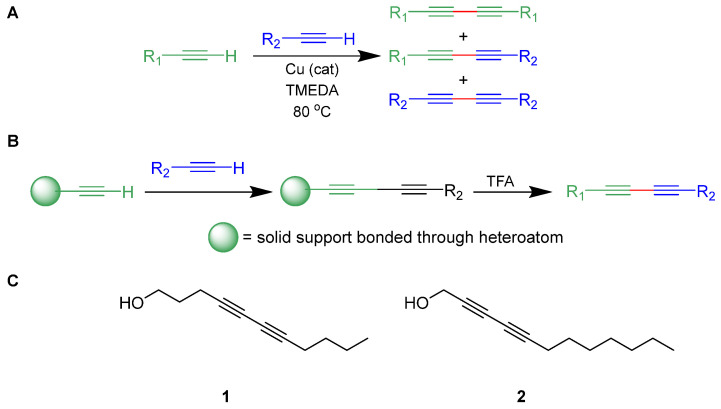
(**A**) Generalized Glaser–Hay reaction of terminal alkynes; (**B**) selective solid-supported Glaser–Hay reaction; (**C**) biologically active compounds from a previous study [26].

The Glaser–Hay reaction involves the coupling of two terminal alkynes, and is useful in the generation of large libraries due to the commercial availability of numerous alkynes and the relative synthetic access to these moieties (Figure 1A) [27]. While immobilization [28,29,30] allows for selective preparation of the heterodimer (Figure 1B) on a small scale, traditional synthesis in solution can easily be performed on a large scale, leading to statistical mixtures of two homodimers and a heterodimer. However, with the substrates described, isolation of the heterodimers was facile, and an array of conjugated alkynes was rapidly prepared and purified using the Glaser–Hay reaction in solution. Formation of three different diynes in this manner also allowed for screening of the two homodimers.

These libraries were then screened for activity in decreasing viability of both *Escherichia coli* and *Pseudomonas fluorescens* cultures. Excitingly, several of the heterodimer compounds exhibited bacterial inhibition, and some demonstrated selective activity for one species over the other; none of the homodimers displayed activity. With respect to the activity in *E. coli*, heterodimers harboring between 10 and 12 carbons were most active. Moreover, controls with fully saturated alcohols, monoyne derivatives, and compounds lacking propargylic alcohol were not as active in the viability assays. Ultimately, two diynes (**1** and **2**) were identified to be the most active in inhibiting bacterial growth (Figure 1C). These results identified **1** as *E. coli*-specific targeting and **2** as a general Gram-negative antibiotic inhibiting both *E. coli* and *P. fluorescens*, but not the Gram-positive *M. smegmatis*. With these compounds in hand, we now seek to identify further structural components vital for antibacterial activity and improve upon their efficacy. Moreover, *P. fluorescens*, a major contaminant of water supplies and in food spoilage, is known as a model organism for biofilm growth, allowing us to attempt to identify if any of these compounds, or their derivatives, are capable of disrupting pre-formed biofilms [31].

## 2. Results

### 2.1. Synthesis

Based on the previously observed results of bacterial inhibition by polyyne compounds, we first sought to investigate a more targeted structure–activity relationship study based on **1** and **2**. As prior results indicated 10–12 carbon polyynes with primary alcohols were most efficacious, we limited our target molecules to around this range of carbons. Specifically, we set out to explore the effects of the distance of the alcohol moiety from the alkynyl core of the molecule as well as the effects of secondary alcohols, since all of the previously screened diynes were based on primary alkynol starting materials.

#### 2.1.1. Alkynol Preparation

**Scheme 1:**
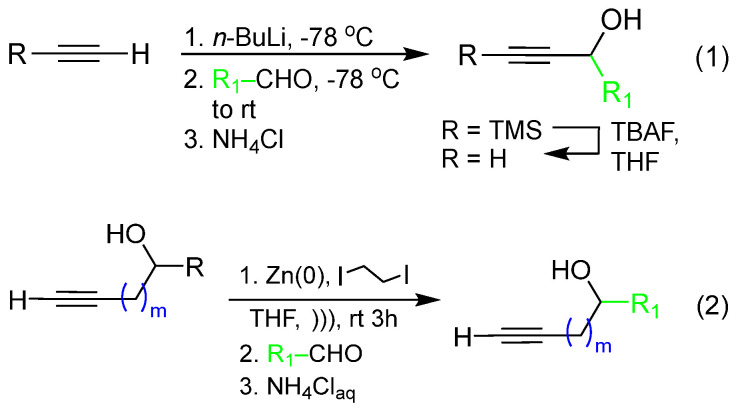
Synthetic routes to propargylic (1) and homopropargylic alcohols (2) used for Glaser–Hay coupling reactions.

Propargylic and homopropargylic secondary alkynols were either commercially available or made by one of two methods shown in (1) and (2), respectively (Scheme 1): (a) direct addition of TMS-acetylide to acetaldehyde, propionaldehyde, butyraldehyde, or valeraldehyde followed by desilylation with TBAF [31] or (b) addition of propargyl zinc bromide [32] to the acetaldehyde, propionaldehyde, and butyraldehyde. All were purified by column chromatography before further use in coupling reactions.

#### 2.1.2. Diyne Synthesis

**Scheme 2:**
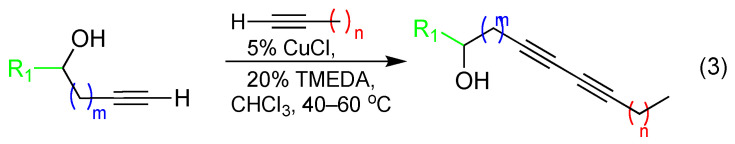

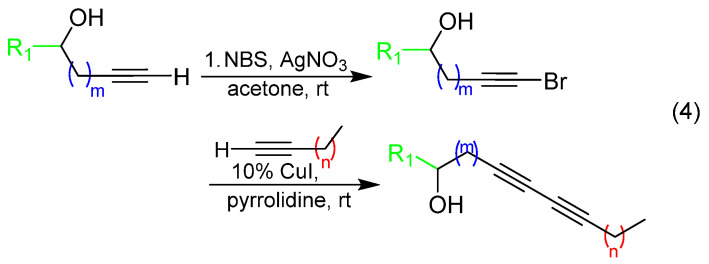
Synthetic procedures for conversion of terminal alkynes to diynes via Glaser–Hay coupling (3) and conversion of terminal alkynes to brominated alkynes, followed by Cadiot–Chadkiewicz alkyne coupling (4).

With alkynol partners in hand, we next sought to construct the diyne library via reaction with variable-chain-length aliphatic terminal alkynes. In order to maximize screening compounds and fully characterize the compounds, we opted to conduct Glaser–Hay reactions in solution to obtain three diyne products: two homodimers and one heterodimer (heterodimer shown in (3), Scheme 2). Given the presence of an alkynol as one coupling partner, these three products were easily separable via column chromatography because of drastic differences in polarities. Based on the previous observations of bioactive compounds harboring 10–12 carbon units, the initial array was constructed around this restriction (Table 1). In several cases, heterodimer yields were low, so the synthetic approach was modified to employ a Cadiot–Chodkiewicz reaction through the preparation of a bromo-alkyne intermediate to help direct reactivity and favor heterodimeric products ((4), Scheme 2) [33,34,35,36]. Diynes were isolated as single components according to TLC analysis and judged to be approximately 95 pure or greater by NMR spectroscopy. ^1^H and ^13^C{^1^H} APT NMR spectra and HRMS data for all diynes are contained in the Supporting Information.

### 2.2. Screening

Upon synthesis and characterization of the diyne array with variable aliphatic tethers and propargylic or homopropargylic units, the compounds were subjected to a variety of bacterial screens to assess bioactivity.

#### 2.2.1. Viability Screens

**Figure 2:**
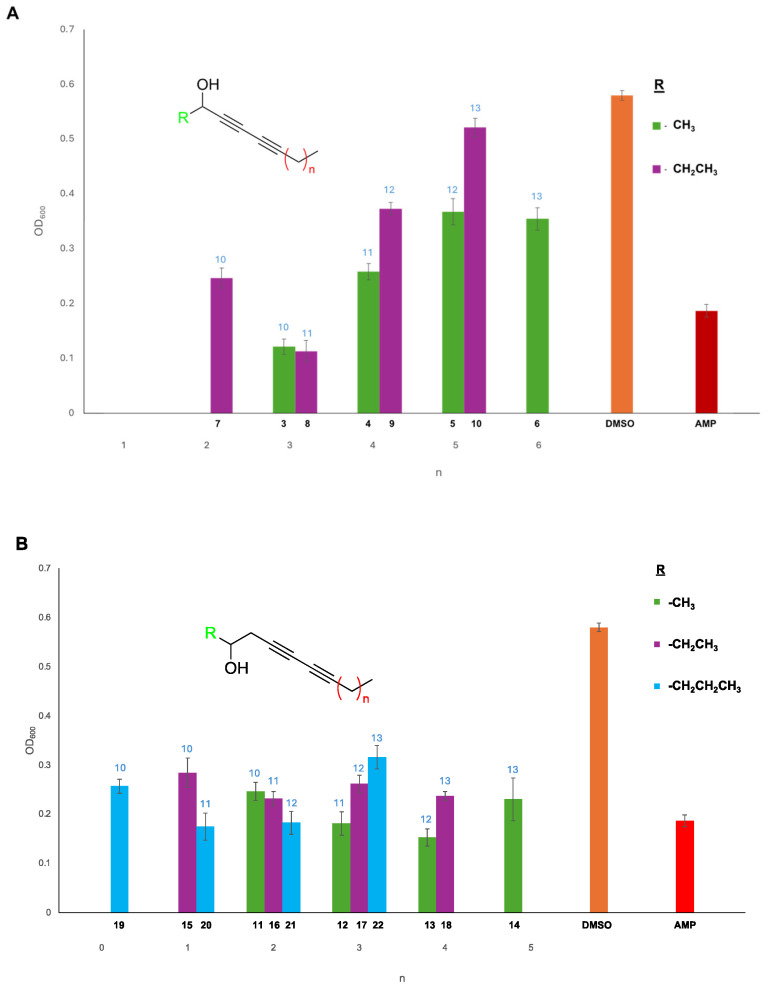
Viability assay of *E. coli* grown in the presence of the secondary propargylic (**A**) and homopropargylic (**B**) diyne arrays. The OD_600_ was measured over 24 h to monitor bacterial growth, and the data were reported at the 8 h measurement time. All compounds were screened in triplicate, and plates were normalized to the DMSO (orange) positive control to allow comparison. Several compounds possessed greater bioactivity than a known *E. coli* inhibitor ampicillin (red). Variation in aliphatic chain length from the terminal alkyne (x-axis) and the alkynol component (R; color) confirmed that compounds ranging from 10 to 13 carbon units were optimal for bioactivity. Standard deviations of the measurements were generated from the replicates in Excel and plotted with the data. The number above the bar (light-blue font) indicates the number of carbon units in the compound.

Initial screens were based on the ability of the compounds to inhibit bacterial growth and survival. These were performed in both *E. coli* and *P. fluorescens* cultures via inoculation at OD_600_ of 0.1 in the presence of the compound and appropriate positive (DMSO) and negative (ampicillin) controls. Each experimental condition was performed in triplicate in a 96-well plate. Cultures were shaken and incubated at 37 °C for 24 h with growth measured spectroscopically at 600 nm in 15 min intervals on a BioTek Microplate Reader (Agilent, Santa Clara, CA, USA) to generate growth curves. After normalization between plates and subtraction of the background, the culture viability was compared after 8 h of growth to determine compound efficacy (Figure 2). All compounds were screened in triplicate to control for experimental variability and allow a statistical determination of the standard deviation. Compounds exhibiting better inhibition than ampicillin were subsequently screened at lower concentrations to better characterize their potency. These screens allowed for the ability to probe the effect of carbon length and secondary alcohol positioning on the viability of various bacteria. These results further confirm the dependence of antibiotic potency on carbon units, with 10–13 being optimal.

#### 2.2.2. Biofilm Disruption Screens

**Figure 3:**
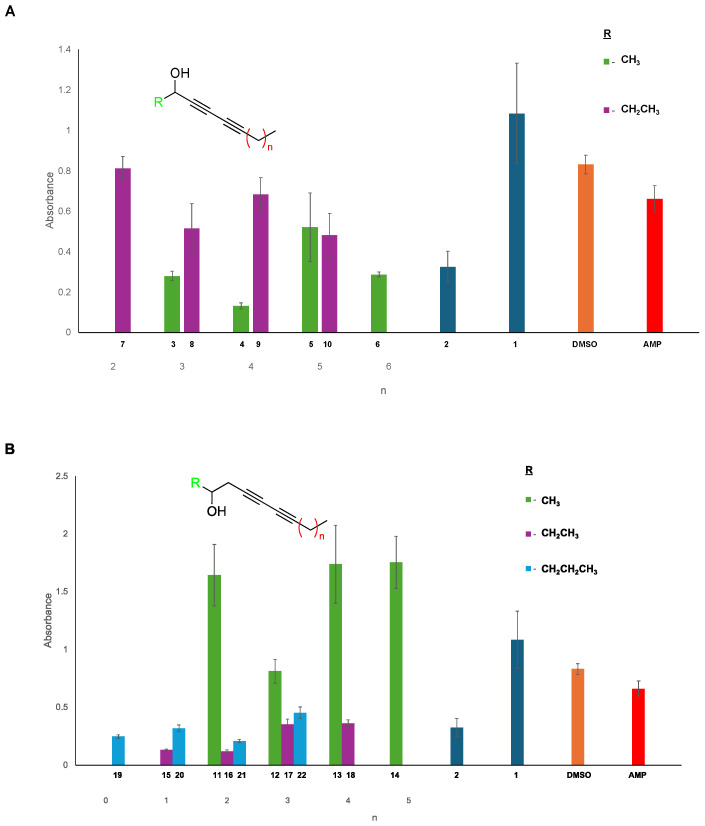
Biofilm disruption assay of *P. fluorescens* in the presence of the secondary propargylic (**A**) and homopropargylic (**B**) diyne arrays. Biofilms were grown and compounds were screened in quintuplicate to minimize error. Following incubation with the compound, the remaining biofilm was stained with crystal violet, absorbances were measured at 550 nm, and plates were normalized to the DMSO (orange) positive control to allow comparison. Previous hits from our prior publication are also included for comparison (dark blue). Decreased absorbance indicates the presence of less biofilm. While not an excellent biofilm disruptor, compounds were compared to ampicillin (red) as a benchmark. Variation in aliphatic chain length from the terminal alkyne (x-axis) and the alkynol component (R; color) indicated that several hit compounds in the homopropargylic series were especially effective in the disruption of biofilm. Standard deviations of the measurements were generated from the replicates in Excel and plotted with the data.

In addition to viability, we became interested in screening these compounds for their ability to disrupt a pre-formed biofilm. In this assay, a *P. fluorescens* biofilm was grown for 24 h in each well of a 96-well plate. The media were then removed and replaced with fresh media supplemented with a library member or an appropriate control. Each assay was conducted in quintuplicate to minimize well-to-well variability. After growth for 24 h in the presence of compound, the media were removed and the wells were washed and stained with crystal violet according to the literature protocols [37]. After solubilization of the stain, absorbance was read at 550 nm to measure the amount of biofilm remaining in each well (Figure 3). Decreased absorbance values indicate the presence of less crystal violet stain and thus less biofilm. Several compounds demonstrated potent biofilm disruption potential, especially within the homopropargylic series (Figure 3B). There was a significant difference in activity within this series between the methyl substitution and the ethyl or propyl derivatives.

## 3. Discussion

Previous work identified the asymmetrical coupling product of 1-hexyne with 4-pentyn-1-ol (**1**) to be a specific inhibitor of *E. coli* over *P. fluorescens* (a Gram-negative bacterium) and *M. smegmatis* (a Gram-positive bacterium) [26]. A second compound afforded by the asymmetrical coupling of 1-nonyne and propargyl alcohol to yield **2** possessed broad-spectrum activity against Gram-negative bacteria but had little effect on Gram-positive bacteria. Additional compounds were found to only have activity against *P. fluorescens*. Each of the bioactive compounds exhibited a K_i_ of ~0.5 µM. While some work was performed to identify the necessity of a diyne versus a single or no alkynyl unit, internal and terminal diynes, and the specific carbon length, no other structural variations were probed. Moreover, all compounds were primary alcohols, and increased substitutions and distances of the polar hydroxyl group from the diyne unit were not examined.

Based on the prior identification of *E. coli* and *P. fluorescens* small-molecule inhibitors harboring a diyne core, a new array of compounds have been successfully synthesized to better understand the key characteristics necessary for bioactivity and to potentially increase the potency of the previously identified hit compounds. While both the hydroxyl group and the aliphatic chain have been previously demonstrated to be important structural components, previous work has not focused on the alcohol functionality, and thus it became the basis of this study. In particular, we focused the library on the substitution and distance of the hydroxyl moiety from the alkynyl core. This is based on both previous results and structural similarities to other biologically active natural products containing polyynes. In order to access more diynes, a solution-based Glaser–Hay synthetic route was adopted, allowing for the isolation of homocoupled alkynes and the asymmetrical diynes from heterocoupling from single reactions. Due to the statistical production of multiple products, a wide range of yields from 17 to 91% of purified diynes were observed. It was also observed that for diynes **3**–**22**, the alkynol precursors were far less stable than the diynes themselves, which were stable over the course of at least 60 days at ~4 °C. Therefore, the alkyne coupling should be performed promptly after isolation of the alkynols.

Once prepared, the array of secondary alcohol diynes were first screened for their ability to impact *E. coli* viability in a growth inhibition assay (Figure 2). All compounds were screened in triplicate and absorbance data was normalized to DMSO controls to allow plate-to-plate comparisons. While all compounds between 10 and 13 carbons in length exhibited some activity, the homopropargylic series (**11**–**21**) did not have any inhibitory effect greater than the previously identified compounds. Compounds possessing 14 carbons in length lost all bioactivity in this assay, re-confirming the structural requirement of a specific chain length that was previously observed. Also, homodimers screened displayed no potent activity.

Within the propargylic series, both compounds **3** and **8** maintained activity comparable to the previous hit compounds. Dilution assays indicated they have a K_i_ of 0.52 µM and 0.61 µM, respectively (for reference, **1** had a K_i_ of 0.63 µM). These compounds are both generated via the coupling of 1-hexyne with the methyl or ethyl propargylic alkyne, indicating that the length of aliphatic units proceeding the diyne moiety is a major contributor to bioactivity, and the hydrophilic propargylic end of the compound is more tolerable to modification. We hypothesize that this may be due to interaction of the hydrophobic region with a specific protein pocket or potentially integration into a membrane. Within the propargylic series, much more activity was lost at longer chain lengths with the 13-carbon diyne, **10**, essentially being inactive (Figure 2). Unlike previously discovered compounds, **3** and **8** did have slight activity in *P. fluoresens* growth assays (see Supporting Information), but their effects were minimal relative to other previously discovered compounds that were more specific to only *E. coli* or *P. fluorescens*. Additionally, from the *P. fluorescens* screens, none of the compounds from either series demonstrated a significant improvement over the previously discovered primary alcohol derivatives (see Supporting Information).

However, given the demonstrated ability of some of the compounds to impede the viability of *P. fluorescens*, we next explored their ability to formally disrupt *P. fluorescens* biofilms once formed. Biofilm disruption is extremely beneficial as biofilms are adaptive responses of bacteria that make them more resistant to antibiotic intervention based on their complex structure that decreases their permeability. Moreover, biofilm formation is especially problematic in the field of human healthcare, so functional disruptors may find widespread application. Based on a well-established biofilm disruption assay employing crystal violet staining, *P. fluorescens* biofilms were grown in a 96-well format with compounds added to each well from initial 50 mg/mL stocks. After a 24 h incubation, the biofilms were stained to ascertain their presence and integrity. Interestingly, several compounds from each series exhibited the ability to disrupt biofilm formation (Figure 3).

**Figure 4:**
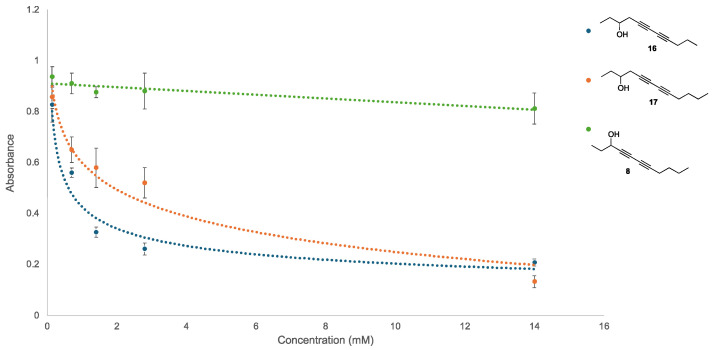
Dose–response curve for key disruptors of established biofilms. Compound **8** was able to suppress *E. coli* growth in other assays but had little effect on *P. fluorescens* biofilm disruption and serves as a control. Compound **16** has the greatest potential as a biofilm disruptor but interestingly was only a moderate effector of *P. fluorescens* viability in previous assays. Increasing the aliphatic chain length by one carbon unit in compound **17** retains disruption ability, albeit at lower levels. Increased biofilm disruption results in lower absorbance values, and all experiments were performed in pentaplex to minimize well-to-well variability.

From the compounds containing a propargylic alcohol, diyne **4** exhibited significant activity in disrupting the formed biofilm, and from the series with homopropargylic alcohols, **15** and **16** were found to have similar activity. It is interesting to note that these compounds were not the most potent in viability screens (see Supporting Information). This suggests that biofilm disruption is not purely a result of antibiotic activity, but other factors are also in play. Dose–response curves for these compounds were generated, and the K_i_ of the compounds were found to be 85.8 μM for **15** and 71.4 μM for **16** (Figure 4). Of the compounds screened, **16** was the most potent inhibitor of biofilm formation. These compounds perform better than the previously reported primary alcohol viability hits and even compounds within this library that have higher efficacy on *P. fluorescens* growth and viability. As illustrated in this dose–response curve, increasing the aliphatic chain by a single carbon retains activity but is less effective. This result demonstrates the importance of an iterative screening of small compound libraries to best understand structure–activity relationships. Interestingly, within the propargylic series, the methyl derivatives were more active than active ethyl derivatives, while for the homopropargylic series, the ethyl and propyl derivatives were most active, and the methyl derivatives actually facilitated biofilm growth. To assess broad biological activity, the hit compounds were screened in an MTT viability assay in mammalian HeLa cell lines and found to have no effect (see Supporting Information). This is in accordance with similar screens performed with the primary alcohol derivatives not altering mammalian cell viability. Unlike the viability assays, no evident carbon length trends were observed as a determinant of disruption capacity, and activity is much more closely linked to the alkynol component than the aliphatic component. The differential activity of compounds that diverge by only a single carbon unit demonstrates the necessity of this systematic and methodical preparation of compound libraries to best identify hits and ultimately understand their mechanism of action. We hypothesize that the semi-amphipathic nature of these compounds may provide some mechanism to intercalate within the biofilm and disrupt its stability; however, continuing experiments are underway to more concretely identify the underlying source of bioactivity.

## 4. Materials and Methods

### 4.1. General Procedure A: Glaser–Hay Reaction 

Copper (I) chloride (0.3 mmol, 0.05 equiv.) and *N*,*N*,*N*′,*N*′-Tetramethylethylenediamine (1.2 mmol, 0.2 equiv.) were added to 10 mL of CHCl_3_ and stirred for 10 min. The 1-alkyne (7.8 mmol, 1.3 equiv.) and alkynol (6.0 mmol, 1.0 equiv.) were then added to the solution in quick succession and heated to 60 °C for 18–24 h under oxygen. The reaction was then diluted with CHCl_3_ (ca. 10 mL), washed with a saturated solution of aqueous NH_4_Cl (ca. 3 × 15 mL), and dried with magnesium sulfate. Solvents were removed in vacuo, and the resulting product was purified via column chromatography on silica gel (20–30% ethyl acetate in hexanes). Molar quantities and isolated yields for pure compounds are shown in Table 1, as well as the Supporting Information [27].

For reactions with 1-pentyne, Glaser–Hay reactions were run for 48 h at 40 °C due to the volatility of the 1-alkyne. If starting alkynol was still present via TLC, the recovered crude material was resubjected to reaction conditions.

### 4.2. General Procedure B: Synthesis of Bromoalkynol Intermediates 

AgNO_3_ (0.24 mmol, 0.08 equiv.) was added to 6.0 mL of dry acetone and stirred for 5–10 min. *N*-Bromosuccinimide (3.6 mmol, 1.2 equiv.) and the alkynol (3.0 mmol, 1.0 equiv.) were then added to the reaction flask and stirred for 2 h at room temperature. The reaction was then diluted with 5 mL of hexanes, washed with H_2_O (3 × 5 mL), and the organic phase dried with MgSO_4_. Excess solvent was removed in vacuo; the crude bromoalkynol was sufficiently pure for use in subsequent Cadiot–Chodkiewicz reactions [35].

### 4.3. General Procedure C: Cadiot–Chodkiewicz Reaction 

Copper (I) iodide (0.16 mmol, 0.1 equiv.) was added to a stirred solution of pyrrolidine (5 mL) and 1-bromo-1-alkynol (1.6 mmol, 1.0 equiv.) in a 15 mL screw-capped pressure vessel, and the solution was cooled to −40 °C using a dry-ice/CH_3_CN bath. Excess propyne or 1-butyne (ca. 3–4 mL) was then condensed into the cold vessel using a gas dispersion tube. If the reaction turned blue or turquoise, hydroxylamine hydrochloride was added to reduce the copper catalyst. The reaction was warmed to room temperature and stirred for 1 h. The reaction was then carefully hydrolyzed with aqueous NH_4_Cl (ca. 10 mL), extracted with ether (3 × 15 mL), and dried with MgSO_4_. Excess solvent was removed in vacuo, and the products were purified using column chromatography on silica gel (20% ethyl acetate in hexanes) [38].

Spectral data as well as ^1^H and ^13^C [^1^H] NMR spectra of diynes **3**–**22** are included in the Supporting Information.

### 4.4. Bacterial Viability Assay

Luria–Bertani (LB) media were inoculated with either *Escherichia coli* (Novagen BL21 (DE3) strain) or *Pseudomonas fluorescens* (AddGene) and incubated at 37 °C for 24 h. The optical density of the culture was assessed with a Genesys Spectrophotometer (Thermo-Fisher, Watlham, MA, USA) at 600 nm. LB media were added to dilute the cultures to an OD_600_ value of 0.10 to employ in the assay. Then, 148 μL of screening culture was added to each well of a 96-well plate, and 2 μL of test compound (50 mg/mL) or DMSO (positive control) or ampicillin (50 mg/mL; USB Corporation (Cleveland, OH, USA); 99% purity; negative control) was added to a final well volume of 150 μL, and each compound was tested in triplicate. The plate was covered with an adhesive film for culture plates to minimize evaporation. Absorbance readings (OD_600_) were taken every 15 min over the course of 24 h using a Synergy HT Microplate Reader (Agilent, Santa Clara, CA, USA). The microplate reader was allowed to shake and incubate at 37 °C. The data were exported and analyzed in Microsoft Excel^TM^.

### 4.5. Biofilm Formation Assay 

This assay was adapted from the literature, and a culture of *P. fluorescens* bacteria was used to inoculate LB media and incubated over a period of 24 h at 37 °C. The culture was then diluted 1:20 in M63 minimal media supplemented with magnesium sulfate (0.10 mM), glucose (10.5 mM), and casamino acids (4.8 g/L). The minimal media (180 μL) were added to each well of a 96-well plate. A protective adhesive film was added to minimize evaporation. The plate was incubated at 37 °C for 24 h. After incubation and growth of the biofilm, the media were replaced, and test compound (10 μL; 50 mg/mL) or DMSO (10 μL) or ampicillin (10 μL; 50 mg/mL) was added to each well. To minimize well-to-well variability, each compound was tested in quintuplicate. Additional M63 minimal media were added to a final volume of 200 μL. The plate was incubated for another 24 h at 37 °C. After incubation, the wells were rinsed three times with DI water and dried. The wells were then treated with 50 μL of 0.1% crystal violet solution and shaken for 15 min. The plate was rinsed three more times with DI water and inverted to dry overnight. To the dry plate, 125 μL of 30% acetic acid was added, followed by incubation for 15 min to solubilize the stained biofilm. The solubilized crystal violet solution was transferred to a clean plate, and the absorbance was measured at 550 nm using the SpectraMax iD3 (Molecular Devices, San Jose, CA, USA). The data were exported and analyzed in Microsoft Excel^TM^ [37].

## 5. Conclusions

Overall, further clarification on the bioactivity of diyne molecules was elucidated through the investigation of secondary alcohols. While the substitution of the alcohol does not appear to have significant effect on the efficacy of the compounds, the new series does demonstrate a similar trend in the dependence of the number of carbon units to maintain antibiotic potential. More excitingly, several of the new compounds disrupt pre-formed biofilms at micromolar concentrations. These compounds may have broader medical relevance, and current investigations are underway to conduct further structure–activity relationship assays to increase their potency and potentially uncover their mechanisms of action. The elucidation of new antibiotics with novel mechanisms of action is crucial given the rise of antibiotic resistance, and this work represents the early stages of leveraging polyyne libraries towards this goal. Moreover, the systematic screening of well-defined libraries has been demonstrated to be valuable as significant differences in antibiotic potential have been observed with the simple addition or subtraction of a methylene unit. Overall, this research provides a new core structure for further investigations with ultimate utility towards the disruption of pre-established biofilms.

## Figures and Tables

**Table 1:** Unsymmetric diynes synthesized for biological screening.

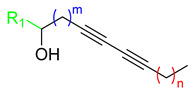					
Compound	R_1_	m	n	# C	Yield ^1^
**3**	CH_3_	0	3	10	28%
**4**	CH_3_	0	4	11	17%
**5**	CH_3_	0	5	12	19%
**6**	CH_3_	0	6	13	66% ^2^
**7**	CH_2_CH_3_	0	2	10	33%
**8**	CH_2_CH_3_	0	3	11	31%
**9**	CH_2_CH_3_	0	4	12	26%
**10**	CH_2_CH_3_	0	5	13	44%
**11**	CH_3_	1	2	10	55%
**12**	CH_3_	1	3	11	91%
**13**	CH_3_	1	4	12	43%
**14**	CH_3_	1	5	13	73%
**15**	CH_2_CH_3_	1	1	10	37% ^2^
**16**	CH_2_CH_3_	1	2	11	32%
**17**	CH_2_CH_3_	1	3	12	62%
**18**	CH_2_CH_3_	1	4	13	67%
**19**	CH_2_CH_2_CH_3_	1	0	10	47% ^2^
**20**	CH_2_CH_2_CH_3_	1	1	11	48% ^2^
**21**	CH_2_CH_2_CH_3_	1	2	12	89%
**22**	CH_2_CH_2_CH_3_	1	3	13	58%

^1^ Yields are based on a statistical probability of 33% maximal yield of the heterodimer using the alkynol as the limiting reagent. ^2^ Compound synthesized via a Cadiot–Chodkiewicz reaction of a halo-alkyne.

## Data Availability

The original contributions presented in this study are included in the article/Supplementary Materials. Further inquiries can be directed to the corresponding author(s).

## Supplementary Materials

The following supporting information can be downloaded at: https://www.mdpi.com/article/10.3390/molecules29245945/s1. Graphs of *P. fluorescens* viability screens with diynes **3**–**22**. Experimental procedures and characterization data for compounds **3**–**22** as well as ^1^H and ^13^C{^1^H}APT NMR spectra for compounds **3**–**22**.
