# Supplementary material for: Elucidation of Antimicrobials and Biofilm Inhibitors Derived from a Polyacetylene Core
Source: Molecules. 2024 Dec 17;29(24):5945. doi: 10.3390/molecules29245945 (PMC11677313; doi:10.3390/molecules29245945)
Supplement: Supplementary file 1 [file molecules-29-05945-s001.zip › molecules-3325409-supplementary.pdf]

Elucidation of Antimicrobials and Biofilm Inhibitors Derived from a Polyacetylene Core

Tyler L. Skeen, Rebekah L. Gresham, Katherine A. Agamaite, Olivia M. Molz, Isabelle F. Westlake, Sage M. Kregenow, Al K. Romero, Brian M. Flood, Lauren E. Mazur, Robert J. Hinkle * and Douglas D. Young *

Department of Chemistry, William & Mary; Williamsburg, VA 23185, USA

* Correspondence: rjhink@wm.edu (R.J.H.); dyoung01@wm.edu (D.D.Y.); Tel.: +1-757-221-1501 (R.J.H.); +1-757-221-2359 (D.D.Y.)

Table of Contents

General Experimental Information 2

Preparation of Stock solutions and dilutions for bioassays: 2

Figures S1, S2 and S3: Graphical representations of *P. fluorescens* or HeLa cell viability with propargylic and homopropargylic alcohols. 3

Preparation of Diynes and Characterization Data 5

Preparation of Alkynols and Brominated Alkynols, and Characterization Data. 10

^{1}H and $^{13}C\{^{1}H\}$ APT NMR Spectra for Diynes 3-22, and Representative Spectra of a Brominated Alkynol. 12

General Experimental Information

Reactions for the synthesis of alkynols were carried out under argon whereas Glaser-Hay couplings were carried out under an oxygen atmosphere and all yields reported based on the alkynol as limiting reagent and the generalized assumption that the maximum expected yield of heterodimer is 33%. Therefore, based upon 0.60 mmol of alkynol, an isolated yield of 0.20 mmol of heterodimer equals a 100% yield. All reagents were purchased commercially unless otherwise noted. Propargylic alkynols (1-butyn-3-ol and 1-pentyn-3-ol), 4-pentyn-2-ol, copper (I) chloride, *n*-butanal, 1,2-diiodoethane, zinc (0) dust, 1-octyne, and 1-heptyne were purchased from Oakwood chemical. 5-hexyn-3-ol, 1-butyne, and 1-propyne were purchased from GFS. Propargyl bromide, *N,N,N',N'* – tetramethylethylenediamine, and copper (I) iodide were purchased from Acros Chemical. *N*-Bromosuccinimide was purchased from Sigma Aldrich. Silver nitrate was purchased from Baker Chemical. Flash column chromatography was performed using 40-75 μm silica gel (200 x 400 mesh). Thin-layer chromatography was conducted using general-purpose TLC plates with silica gel on glass. Visualization of products was accomplished via potassium permanganate or *p*-anisaldehyde staining solution and heating until spots resolved. ^{1}H NMR spectra were recorded on a 400 MHz FTNMR spectrometer and standardized to the TMS peak at 0.00 ppm. Proton-decoupled ^{13}C NMR (attached proton test, APT) were recorded at 100 MHz and used $CHCl_3$ as an internal standard at 77.0 ppm. Positive peaks in the APT spectra correspond to quaternary or methylene (CH_2) carbons whereas negative peaks correspond to methine (CH) and methyl (CH_3) carbons. All peaks are reported in parts per million (ppm). High resolution mass spectral analyses were conducted by the COSMIC center at Old Dominion University. The analyses were completed using positive-ion mode electrospray ionization with a hybrid FTMS instrument.

Preparation of Stock solutions and dilutions for bioassays: 20-30 mg of each product was added to an Eppendorf tube. Dimethyl Sulfoxide (DMSO) was added to afford a stock of 50mg/mL of each product. Each dilution 1:10, 1:100 was created with serial dilutions for final concentrations of 5 and 0.5 mg/mL respectively of each product. Each compound stock solution was stored in a -26 ̊C freezer.

Mammalian Viability Assay: This procedure follows the protocol outlined by the CellQuanti-MTT Cell Viability Assay Kit (VWR). In triplicate, confluent HeLa cells (AddGene) in a 96-well plate were treated with compounds at the working concentration of 5mg/mL for a final DMSO concentration of 5%. The treated plate was incubated (37°C, 5% CO_2) overnight. Then, the reagent solution (15 µL per 80 µL of DMEM) were added to each aspirated well. The plate was then allowed to incubate at 37 °C for 4 h. Solubilizer (100 µL) was added to each well, and allowed to shake gently at 37°C overnight. The next day an the absorbance was measured at 570 nm on a Synergy HT Microplate

reader. Values were compared to a positive control containing only cells and media, and a negative control where cells had been treated with a bleach solution.

Figures S1, S2 and S3: Graphical representations of *P. fluorescens* or HeLa cell viability with propargylic and homopropargylic alcohols.

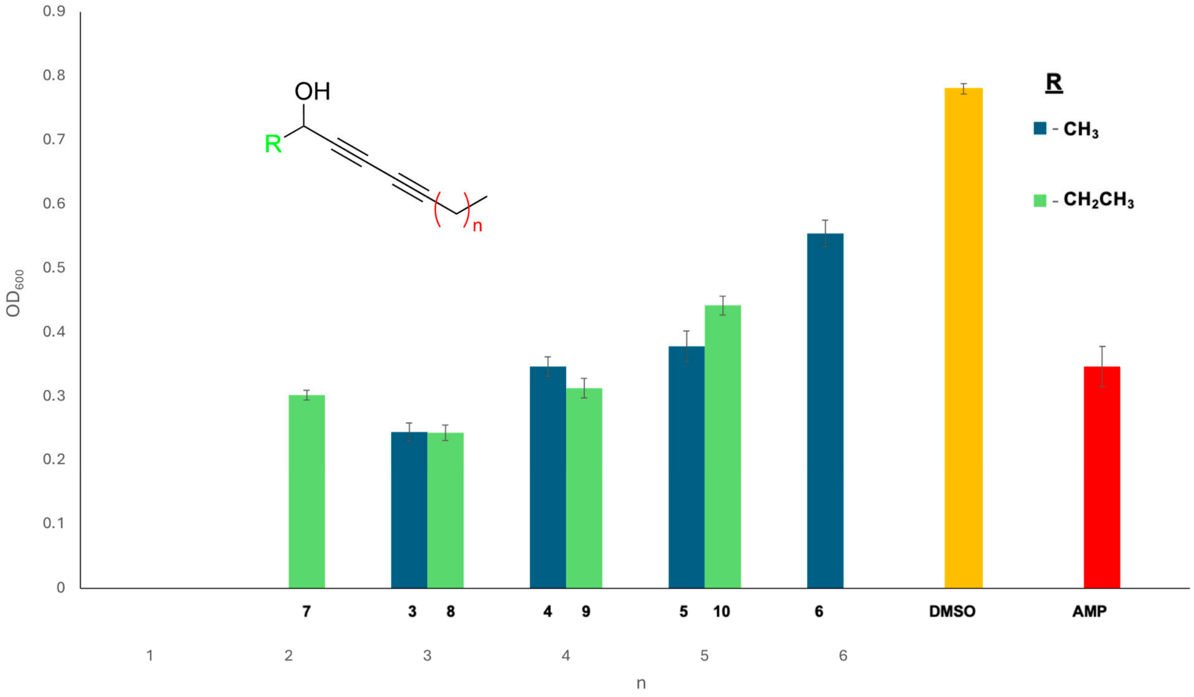

Figure S1. *P. fluorescens* viability screen with propargylic alcohols.

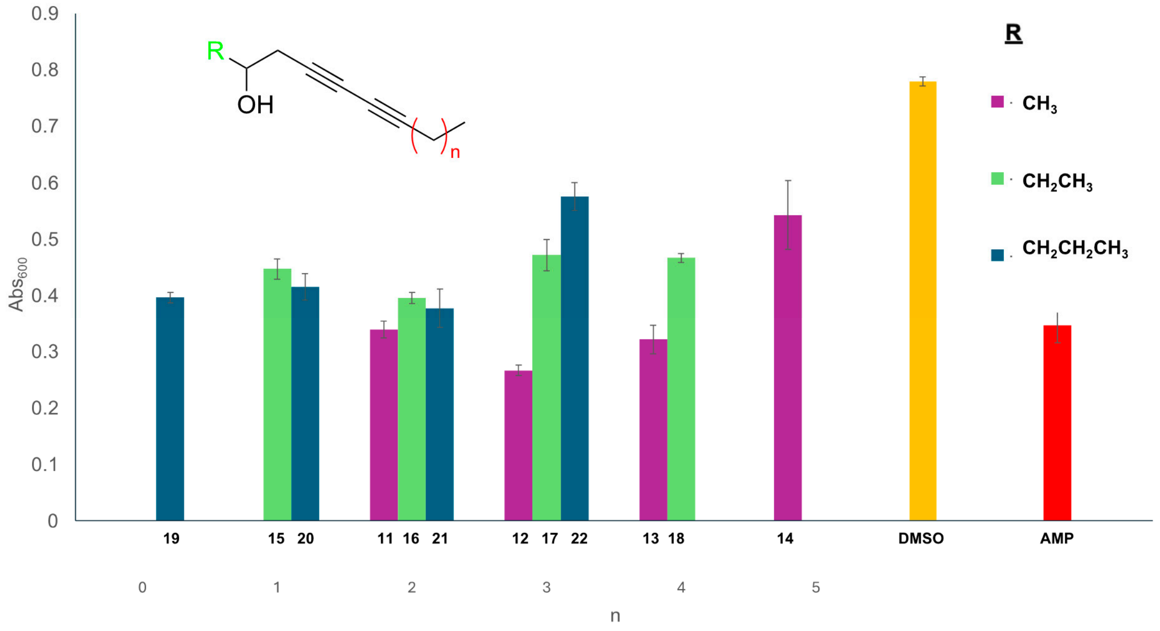

Figure S2. *P. fluorescens* viability screen with homopropargylic alcohols.

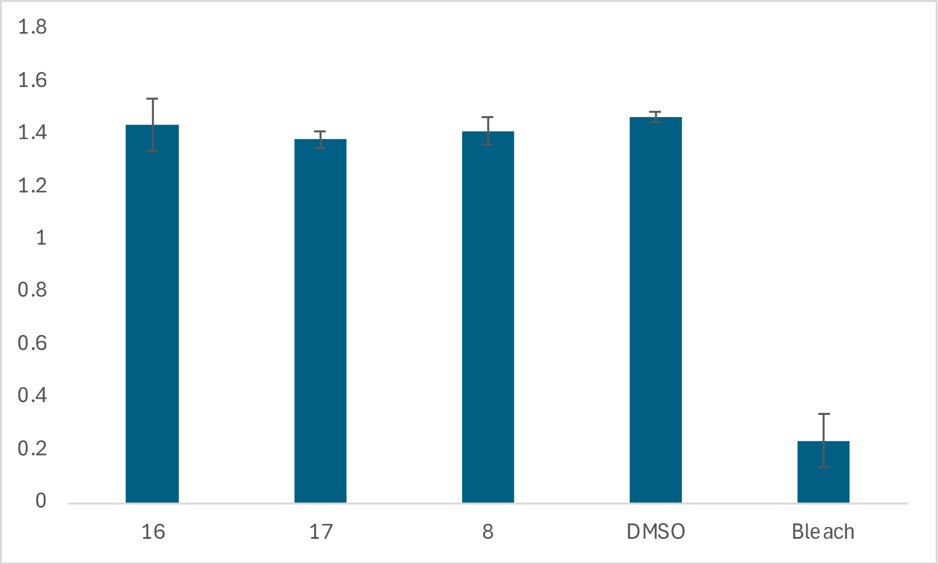

Figure S3. MMT cell viability assay with HeLa cells of lead compounds. DMSO was employed as a positive control and bleach as a negative control. Viability assays were performed in triplicate and demonstrated no significant viability loss of the mammalian cells in the presence of alkyn-ol compounds.

Preparation of Diynes and Characterization Data

Deca-3,5-diyn-2-ol (3) was prepared using general procedure **A** to afford 60 mg (22%) of product from 6.0 mmol of 3-butyn-2-ol; light yellow oil; R_f = 0.65 (30% ethyl acetate in hexanes). ^{1}H NMR (400 MHz, $CDCl_3$) δ: 0.91 (t, J = 7.2 Hz, 3H); 1.39-1.56 (m, 7H); 1.82 (s, 1H); 2.29 (dt, 2H, J_1 = 6.9 Hz, J_2 = 0.8 Hz); 4.57 (p, J = 6.2 Hz, 1H). ^{13}C{^{1}H} NMR (100 MHz, $CDCl_3$) δ: 13.5, 18.9, 21.9, 24.1, 30.1, 58.7, 64.2, 69.2, 77.1, 81.9. HRMS (ESI) m/z $[M + Na]^+$ calcd for $C_{10}H_{14}ONa$ 173.0937, found 173.0940.

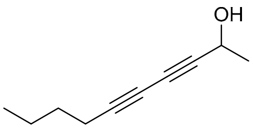

3-butyn-2-ol + 1-hexyne
deca-3,5-diyn-2-ol
Chemical Formula: $C_{10}H_{14}O$
Molecular Weight: 150.22

Undeca-3,5-diyn-2-ol (4) was prepared using general procedure **A** to afford 57 mg (17%) of product from 6.0 mmol of 3-butyn-2-ol; light yellow oil; R_f = 0.56 (30% ethyl acetate in hexanes). ^{1}H NMR (400 MHz, $CDCl_3$) δ: 0.90 (t, J = 7.0 Hz, 3H); 1.24-1.41 (m, 4H); 1.46 (d, J = 14.1 Hz, 3H); 1.50-1.57 (m, 2H); 1.79 (s, 1H); 2.28 (dt, J = 0.8, 7.0 Hz, 2H); 4.56 (q, J = 6.7 Hz, 1H). ^{13}C{^{1}H} NMR ($CDCl_3$) δ: 13.9, 19.2, 22.1, 24.1, 27.8, 30.9, 58.7, 64.2, 69.2, 77.1, 82.0. HRMS (ESI) m/z $[M + Na]^+$ calcd for $C_{11}H_{16}ONa$ 187.1093, found 187.1094.

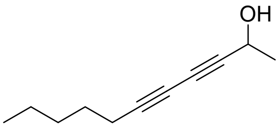

undeca-3,5-diyn-2-ol
Chemical Formula: $C_{11}H_{16}O$
Molecular Weight: 164.24800
3-butyn-2-ol + 1-heptyne

Dodeca-3,5-diyn-2-ol (5) was prepared using general procedure **A** to afford 66 mg (18%) of product from 6.0 mmol of 3-butyn-2-ol; light yellow oil; R_f = 0.41 (20% ethyl acetate in hexanes) ^{1}H NMR (400 MHz, $CDCl_3$) δ: 0.89 (t, J = 6.9 Hz, 3H), 1.32 (m, 6H), 1.46 (d, J = 6.7 Hz, 3H), 1.49-1.56 (m, 2H), 1.84 (br s, 1H), 2.28 (t, J = 7.0 Hz, 2H), 4.56-4.57 (m, 1H). ^{13}C{^{1}H} NMR (100 MHz, $CDCl_3$) δ: 14.0, 19.2, 22.5, 24.0, 28.1, 28.5, 31.2, 58.7, 64.2, 69.2, 77.1, 81.9. HRMS (ESI) m/z $[M + Na]^+$ calcd for $C_{12}H_{18}ONa$ 201.1250, found 201.1252.

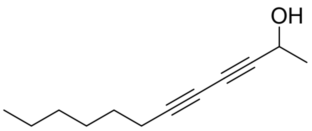

dodeca-3,5-diyn-2-ol
Chemical Formula: $C_{12}H_{18}O$
Molecular Weight: 178.27500
3-butyn-2-ol + 1-octyne

Trideca-3,5-diyn-2-ol (6) was prepared using general procedure **C**, with all reagents and solvents scaled proportionally, to afford 120 mg (66%) of product from 0.94 mmol of 4-bromo-3-butyn-2-ol; light yellow oil; Rf = 0.55 (20% ethyl acetate in hexanes). ^{1}H NMR (400 MHz, $CDCl_3$): 0.88 (t, J = 6.8 Hz, 3H); 1.26-1.40 (m, 8H); 1.46 (d, J = 6.7 Hz, 3H); (1.48-1.56) (m, 2H); 1.96 (s, 1H); 2.27 (t, J= 7.0 Hz, 2H); 4.55 (q, J = 6.7, 1H); ^{13}C{^{1}H} NMR (100 MHz, $CDCl_3$) 14.0, 19.2, 22.6, 24.0, 28.1, 28.7, 28.7, 31.6, 58.6, 64.3, 69.1, 77.1, 81.8. HRMS (ESI) m/z [M + Na]+ calcd for $C_{13}H_{20}ONa$ 215.1406, found 201.1408.

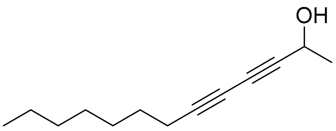

trideca-3,5-diyn-2-ol
Chemical Formula: $C_{13}H_{20}O$
Molecular Weight: 192.30200
3-butyn-2-ol + 1-nonyne

Deca-4,6-diyn-3-ol (7) was prepared using general procedure **A** to afford 98 mg (33%) of product from 6.0 mmol of 1-pentyn-3-ol; R_f = 0.58 (30% ethyl acetate in hexanes). ^{1}H NMR (400 MHz, $CDCl_3$) δ: 1.00 (t, J = 7.4 Hz, 3H); 1.01 (t, J = 7.8 Hz, 3H; 1.57 (sextet, J = 7.3, 2H); 1.68-1.79 (m, 2H); 1.96 (brs, 1H); 2.26 (t, J = 7.0 Hz, 2H); 4.36 (t, J = 6.5 Hz, 1H). $^{13}C\{^1H\}$ NMR (100 MHz, $CDCl_3$) δ: 9.3, 13.4, 21.2, 21.6, 30.7, 64.0, 64.5, 69.9, 76.3, 81.5. HRMS (ESI) m/z $[M + Na]^+$ calcd for $C_{10}H_{14}ONa$ 173.0937, found 173.0939.

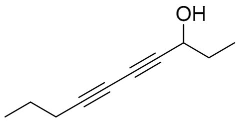

deca-4,6-diyn-3-ol
Chemical Formula: $C_{10}H_{14}O$
Molecular Weight: 150.22100
1-pentyn-3-ol + 1-pentyne

Undeca-4,6-diyn-3-ol (8) was prepared using general procedure **A** to afford 55 mg (31%) of product from 6.0 mmol of 1-pentyn-3-ol; light yellow oil; R_f = 0.474 (20% ethyl acetate in hexanes). ^{1}H NMR (400 MHz, $CDCl_3$) δ: 0.91 (t, J = 7.2 Hz, 3H); 1.01 (t, J = 7.4 Hz, 3H); 1.37-1.46 (m, 2H); 1.48-1.56 (m, 2H); 1.69-1.77 (m, 2H); 1.95 (d, J = 5.5 Hz, 1H); 2.29 (t, J = 6.9 Hz, 2H); 4.36 (q, J = 6.1 Hz, 1H). $^{13}C\{^1H\}$ NMR (100 MHz, $CDCl_3$) δ: 9.3, 13.5, 18.9, 21.9, 30.2, 30.7, 64.1, 64.3, 70.0, 76.2, 81.7. HRMS (ESI) m/z $[M + Na]^+$ calcd for $C_{11}H_{16}ONa$ 187.1093, found 187.1095.

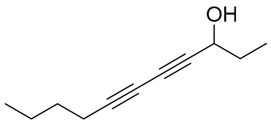

undeca-4,6-diyn-3-ol
Chemical Formula: $C_{11}H_{16}O$
Molecular Weight: 164.24800
1-pentyn-3-ol + 1-hexyne

Dodeca-4,6-diyn-3-ol (9) was prepared using general procedure **A** to afford 91 mg (26%) of product from 6.0 mmol of 1-pentyn-3-ol; light yellow oil; Rf = 0.62 (30% ethyl acetate in hexanes). ^{1}H NMR (400 MHz, $CDCl_3$) δ: 0.90 (t, J = 7.0 Hz, 3H); 1.01 (t, J = 7.4 Hz, 3H); 1.24-1.41 (m, 4H); 1.54 (dt, J = 7.2, 14.5 Hz, 2H); 1.68-1.79 (m, 3H); 2.28 (t, J = 7.0 Hz, 2H); 4.36 (t, J = 6.5 Hz, 1H). $^{13}C\{^1H\}$ NMR (100 MHz, $CDCl_3$) δ: 9.3, 13.9, 19.2, 22.1, 27.8, 30.7, 31.0, 64.1, 64.3, 70.0, 76.2, 81.8. HRMS (ESI) m/z $[M + Na]^+$ calcd for $C_{12}H_{18}ONa$ 201.1250, found 201.1253.

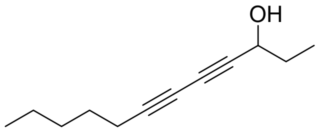

dodeca-4,6-diyn-3-ol
Chemical Formula: $C_{12}H_{18}O$
Molecular Weight: 178.27500
1-pentyn-3-ol + 1-heptyne

Trideca-4,6-diyn-3-ol (10) was prepared using general procedure **A** to afford 170 mg (44%) of product from 6.0 mmol of 1-pentyn-3-ol; light yellow oil; R_f = 0.50 (20% ethyl acetate in hexanes). ^{1}H NMR (400 MHz, $CDCl_3$) δ: 0.89 (t, J = 7.0 Hz, 3H); 1.02 (t, J = 7.4 Hz, 3H); 1.23-1.42 (m, 6H); 1.49-1.57 (m, 2H); 1.69-1.78 (m, 3H); 2.28 (t, J = 7.0 Hz, 2H); 4.36 (q, J = 6.1 Hz, 1H). $^{13}C\{^1H\}$ NMR (100 MHz, $CDCl_3$) δ: 9.3, 14.0, 19.2, 22.5, 28.1, 28.5, 30.7, 31.2, 64.1, 64.3, 70.0, 76.2, 81.7. HRMS (ESI) m/z $[M + Na]^+$ calcd for $C_{13}H_{20}ONa$ 215.1406, found 201.1408.

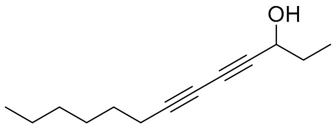

trideca-4,6-diyn-3-ol
Chemical Formula: $C_{13}H_{20}O$
Molecular Weight: 192.30200
1-pentyn-3-ol + 1-octyne

Deca-4,6-diyn-2-ol (11) was prepared using general procedure A to afford 165 mg (55%) of product from 6.0 mmol of 4-pentyn-2-ol; light yellow oil; R_f = 0.38 (30% ethyl acetate in hexanes). ^{1}H NMR (400 MHz, $CDCl_3$) δ: 0.99 (t, J = 7.4 Hz, 3H); 1.27 (d, J = 5.9 Hz, 3H); 1.51-1.60 (m, 2H); 1.88 (d, J = 3.5 Hz, 1H); 2.24 (t, J = 7.0 Hz, 2H); 2.40 (dd, J_1 = 6.5 Hz, J_2 = 17.0 Hz, 1H); 2.47 (dd, J_1 = 5.3 Hz, J_2 = 17.0 Hz, 1H); 3.93-4.02 (m, 1H). ^{13}C{^{1}H} NMR (100 MHz, $CDCl_3$) δ: 13.4, 21.1, 21.7, 22.4, 29.7, 65.0, 66.4, 67.6, 73.4, 78.2. HRMS (ESI) m/z [M + Na]$^+$ calcd for $C_{10}H_{14}ONa$ 173.0937, found 173.0940.

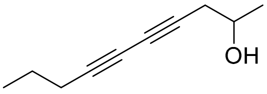

deca-4,6-diyn-2-ol
Chemical Formula: $C_{10}H_{14}O$
Molecular Weight: 150.22100
4-pentyn-2-ol + 1-pentyne

Undeca-4,6-diyn-2-ol (12) was prepared using general procedure **A** to afford 300 mg (91%) of product from 6.0 mmol of 4-pentyn-2-ol; light yellow oil; R_f = 0.52 (30% ethyl acetate in hexanes). ^{1}H NMR (400 MHz, $CDCl_3$) δ: 0.91 (t, J = 7.2 Hz, 3H); 1.27 (d, J = 6.3 Hz, 3H); 1.37-1.55 (m, 4H); 2.10 (s, 1H); 2.26 (t, J = 6.8 Hz, 2H); 2.40 (dd, J_1 = 17.0 Hz, J_2 = 6.5 Hz, 1H); 2.47 (dd, J_1 = 17.0 Hz, J_2 = 5.3 Hz, 1H); 3.96 (sextet, J = 5.4 Hz, 1H). ^{13}C{^{1}H} NMR (100 MHz, $CDCl_3$) δ: 13.4, 18.8, 21.9, 22.4, 29.7, 30.2, 64.9, 66.3, 67.6, 73.4, 78.3. HRMS (ESI) m/z [M + Na]$^+$ calcd for $C_{11}H_{16}ONa$ 187.1094, found 187.1097.

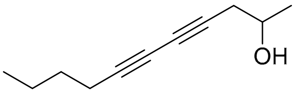

undeca-4,6-diyn-2-ol
Chemical Formula: $C_{11}H_{16}O$
Molecular Weight: 164.24800
4-pentyn-2-ol + 1-hexyne

Dodeca-4,6-diyn-2-ol (13) was prepared using general procedure **A** to afford 153 mg (43%) of product from 6.0 mmol of 4-pentyn-2-ol; light yellow oil; R_f = 0.60 (30% ethyl acetate in hexanes). ^{1}H NMR ($CDCl_3$) δ: 0.90 (t, J = 7.0 Hz, 3H); 1.24-1.41 (m, 7H); 1.53 (dt, J = 7.0, 14.4 Hz, 2H); 1.84 (s, 1H); 3.94-4.01 (m, 1H). ^{13}C{^{1}H} NMR (100 MHz, $CDCl_3$) δ: 13.9, 19.1, 22.1, 22.4, 27.9, 29.8, 31.0, 64.9, 66.4, 67.7, 73.4, 78.4. HRMS (ESI) m/z [M + Na]$^+$ calcd for $C_{12}H_{18}ONa$ 201.1250, found 201.1254.

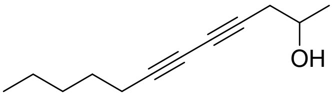

dodeca-4,6-diyn-2-ol
Chemical Formula: $C_{12}H_{18}O$
Molecular Weight: 178.27500
4-pentyn-2-ol + 1-heptyne

Trideca-4,6-diyn-2-ol (14) was prepared using general procedure **A** to afford 280 mg (73%) of product from 6.0 mmol of 4-pentyn-2-ol; yellow oil; R_f = 0.5 (20% ethyl acetate in hexanes). ^{1}H NMR (400 MHz, $CDCl_3$) δ: 0.88 (t, J = 6.9 Hz, 3H), 1.22-1.42 (m, 10H); 1.52 (m, 2H), 2.25 (t, J = 7.0 Hz, 2H); 2.37-2.49 (m, 2H), 3.93-4.00 (m, 1H); ^{13}C{^{1}H} NMR (100 MHz, $CDCl_3$) δ: 14.0, 19.1, 22.4, 22.5, 28.2, 28.5, 29.8, 31.2,

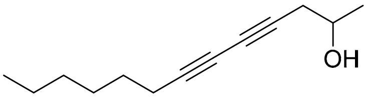

trideca-4,6-diyn-2-ol
Chemical Formula: $C_{13}H_{20}O$
Molecular Weight: 192.30200
4-pentyn-2-ol + 1-octyne

64.9, 66.4, 67.7, 73.3, 78.4. HRMS (ESI) m/z $[M + Na]^+$ calcd for $C_{13}H_{20}ONa$ 215.1406, found 215.1409.

Deca-5,7-diyn-3-ol (15) was prepared using general procedure **C** to afford 90 mg (37%) of product from 1.6 mmol of 6-bromo-5-hexyn-3-ol; yellow oil; R_f = 0.47 (30% ethyl acetate in hexanes). ^{1}H NMR (400 MHz, $CDCl_3$) δ: 0.96 (t, J = 7.4 Hz, 3H); 1.16 (t, J = 7.4 Hz, 3H); 1.50-1.65 (m, 2H); 1.89 (d, J = 5.1 Hz, 1H); 2.27 (q, J = 4.4 Hz, 2H); 2.40 (dd, J_1 = 6.0 Hz, J_2 = 16.5 Hz, 1H); 2.50 (dd, J_1 = 4.9 Hz, J_2 = 17.0 Hz, 1H); 3.66-3.73 (m, 1H); ^{13}C NMR (100 MHz, $CDCl_3$) δ: 9.8, 12.8, 13.3, 27.7, 29.2, 64.4, 67.5, 71.4, 73.6, 79.3. HRMS (ESI) m/z $[M + Na]^+$ calcd for $C_{10}H_{14}ONa$ 173.0937, found 173.0940.

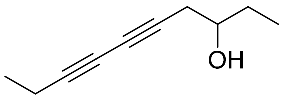

deca-5,7-diyn-3-ol
Chemical Formula: $C_{10}H_{14}O$
Molecular Weight: 150.22100
5-hexyn-3-ol + 1-butyne

Undeca-5,7-diyn-3-ol (16) was prepared using general procedure **A** to afford 106 mg (32%) of product from 6.0 mmol of 5-hexyn-3-ol; yellow oil; R_f = 0.48 (20% ethyl acetate in hexanes). ^{1}H NMR (400 MHz, $CDCl_3$) δ: 0.96 (t, 3H, J = 7.4 Hz); 0.99 (t, 3H, J = 7.4 Hz); 1.50-1.66 (m, 4H); 1.85 (d, 1H, J = 5.1 Hz); 2.24 (t, 2H, J = 7.0 Hz); 2.40 (dd, 1H, J = 6.5, 17.0 Hz); 2.50 (dd, 1H, J = 6.7, 10.6 Hz); 3.66-3.73 (m, 1H); ^{13}C{^{1}H} NMR (100 MHz, $CDCl_3$) δ: 9.8, 13.4, 21.1, 21.7, 27.7, 29.2, 65.1, 67.5, 71.4, 73.5, 78.1. HRMS (ESI) m/z $[M + Na]^+$ calcd for $C_{11}H_{16}ONa$ 187.1094, found 187.1095.

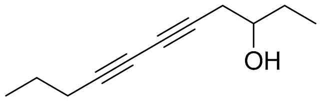

undeca-5,7-diyn-3-ol
Chemical Formula: $C_{11}H_{16}O$
Molecular Weight: 164.24800
5-hexyn-3-ol + 1-pentyne

Dodeca-5,7-diyn-3-ol (17) was prepared using general procedure **A** to afford 222 mg (62%) of product from 6.0 mmol of 5-hexyn-3-ol; light yellow oil; R_f = 0.38 (20% ethyl acetate in hexanes). ^{1}H NMR (400 MHz, $CDCl_3$) δ: 0.91 (t, J = 7.3 Hz, 3H); 0.96 (t, J = 7.4 Hz, 3H); 1.39-1.60 (m, 6H); 1.84 (br s, 1H); 2.26 (t, J = 6.9 Hz, 2H); 2.40 (dd, J = 6.5, 17.1 Hz, 1H); 2.50 (dd, J = 4.7, 17.2, 1H); 3.68 (1H); ^{13}C{^{1}H} NMR (100 MHz, $CDCl_3$) δ: 9.8, 13.5, 18.8, 21.9, 27.7, 29.2, 30.3, 65.0, 67.6, 71.4, 73.4, 78.2. HRMS (ESI) m/z $[M + Na]^+$ calcd for $C_{12}H_{18}ONa$ 201.1250, found 201.1252.

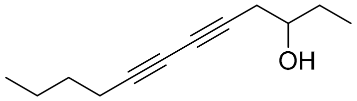

dodeca-5,7-diyn-3-ol
Chemical Formula: $C_{12}H_{18}O$
Molecular Weight: 178.27500
5-hexyn-3-ol + 1-hexyne

Trideca-5,7-diyn-3-ol (18) was prepared using general procedure **A** to afford 257 mg (67%) of trideca-5,7-diyn-3-ol from 6.0 mmol of 5-hexyn-3-ol; light yellow oil; *Rf* = 0.58 (20% ethyl acetate in hexanes). ^{1}H NMR (400 MHz, $CDCl_3$) δ: 0.90 (t, *J* = 7.0 Hz, 3H); 0.96 (t, *J* = 7.4 Hz, 3H); 1.26-1.41 (m, 4H); 1.50-1.62 (m, 4H); 1.82 (d, *J* = 5.1 Hz, 1H); 2.25 (t, *J* = 7.0 Hz, 2H); 2.41 (dd, *J* = 6.7, 17.2 Hz, 2H); 2.50 (dd, *J* = 4.9, 17.0 Hz, 2H); 3.66-3.73 (m, 1H). ^{13}C{^{1}H} NMR (100 MHz, $CDCl_3$): 9.7, 13.8, 19.0, 22.0, 27.6, 27.8, 29.0, 30.9, 65.0, 67.4, 71.3, 73.5,78.0. HRMS (ESI) m/z [M + Na]+ calcd for $C_{13}H_{20}ONa$ 215.1406, found 201.1410.

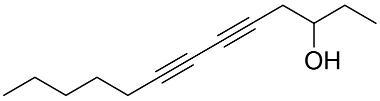

trideca-5,7-diyn-3-ol
Chemical Formula: $C_{13}H_{20}O$
Molecular Weight: 192.30200
5-hexyn-3-ol + 1-heptyne

17. Deca-6,8-diyn-4-ol (19) was prepared using general procedure **C** with all reagents and solvents scaled proportionally, to afford 330 mg (47%) of product from 4.7 mmol of 1-bromo-1-hexyn-4-ol; yellow oil; *R$_f$* = 0.43 (20% ethyl acetate in hexanes) ^{1}H NMR (400 MHz, $CDCl_3$) δ: 0.92 (t, *J* = 7.2 Hz, 3H); 1.23-1.53 (m, 4H); 1.90 (t, *J* = 1.2 Hz, 3H); 3.37 (ddd, *J* = 1.2, 6.3, 17.2 Hz, 1H); 2.47 (ddd, *J* = 1.2, 4.9, 17.0 Hz, 1H); 3.72-3.78 (m,1H). ^{13}C{^{1}H} NMR (100 MHz, $CDCl_3$) δ: 4.1, 13.9, 18.7, 28.1, 38.4, 64.2, 67.6, 69.7, 72.9, 73.7. HRMS (ESI) m/z [M + Na]$^+$ calcd for $C_{10}H_{14}ONa$ 173.0937, found 173.0939.

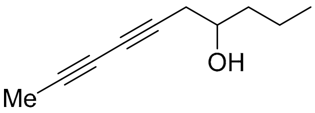

Deca-6,8-diyn-4-ol
Chemical Formula: $C_{10}H_{14}O$
Molecular Weight: 150.22100
hept-1-yn-4-ol + propyne

Undeca-6,8-diyn-4-ol (20) was prepared using general procedure **C** with all reagents and solvents scaled proportionally to afford 140 mg (47%) of product from 1.8 mmol of 1-bromo-1-hexyn-4-ol; light yellow oil; *R$_f$* = 0.44 (20% ethyl acetate in hexanes). ^{1}H NMR (400 MHz, $CDCl_3$) δ: 0.94 (t, *J* = 7.24 Hz, 3H-); 1.16 (t, *J* = 7.4 Hz, 3H); 1.30-1.57 (m, 4H), 1.83 (br s, 1H), 2.49 (dd, *J* = 6.5, 17.0 Hz, 1H) 2.39 (dd, *J* = 4.7, 17.2 Hz, 1H) 3.77 (m, 1H); ^{13}C{^{1}H} NMR (100 MHz, $CDCl_3$) δ:12.9. 13.3, 13.9, 18.8, 28.2, 38.5, 64.4, 67.6, 69.8, 73.7, 70.4. HRMS (ESI) m/z [M + Na]$^+$ calcd for $C_{11}H_{16}ONa$ 187.1093, found 187.1096.

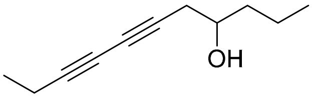

undeca-6,8-diyn-4-ol
Chemical Formula: $C_{11}H_{16}O$
Molecular Weight: 164.24800
hept-1-yn-4-ol +1-butyne

Dodeca-6,8-diyn-4-ol (21) was prepared using general procedure **A** at 40 °C to afford 251 mg (89%) of product from 6.0 mmol of 1-heptyn-4-ol; light yellow oil; *R$_f$* = 0.61 (30% ethyl acetate in hexanes). ^{1}H NMR (400 MHz, $CDCl_3$) δ: 0.94 (t, J = 7.0 Hz, 3H); 0.99 (t, J = 7.2 Hz, 3H); 1.34-1.60 (m, 6H); 2.09 (s, 1H); 2.24 (t, J = 7.0 Hz, 2H); 2.40 (dd, J_1 = 17.2 Hz, J_2 = 6.7 Hz, 1H); 2.50 (dd, J_1 = 17.0 Hz, J_2 = 4.9 Hz, 1H); 3.77 (s, 1H). ^{13}C{^{1}H} NMR (100 MHz, $CDCl_3$) δ: 13.4, 13.9, 18.7,

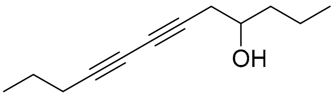

dodeca-6,8-diyn-4-ol
Chemical Formula: $C_{12}H_{18}O$
Molecular Weight: 178.27500
hept-1-yn-4-ol +1-pentyne

21.1, 21.7, 28.1, 38.4, 65.1, 67.5, 69.7, 73.5, 78.0. HRMS (ESI) m/z $[M + Na]^+$ calcd for $C_{12}H_{18}ONa$ 201.1250, found 201.1250.

Trideca-6,8-diyn-4-ol (22) was prepared using general procedure **A** to afford 148 mg (58%) of product from 4.1 mmol of 1-heptyn-4-ol; light yellow oil; R_f = 0.45 (20% ethyl acetate in hexanes). ^1H NMR (400 MHz, $CDCl_3$) δ: 0.89-0.96 (m, 6H); 1.26-1.57 (m, 8H); 1.81 (d, J = 5.1 Hz, 1H); 2.26 (t, J = 7.1 Hz, 2H); 2.49 (dd, J = 5.0, 17.1 Hz, 1H); 2.39 (dd, J = 6.6, 17.2 Hz, 1H); 3.77 (m, 1H); $^{13}C\{^1H\}$ NMR (100 MHz, $CDCl_3$): 13.5, 13.9, 18.78, 18.83, 21.9, 28.2 30.3, 38.5, 65.0, 67.6, 69.8, 73.5, 78.2. HRMS (ESI) m/z $[M + Na]^+$ calcd for $C_{13}H_{20}ONa$ 215.1406, found 215.1410.

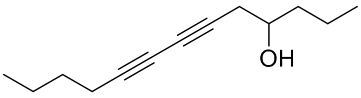

trideca-6,8-diyn-4-ol
Chemical Formula: $C_{13}H_{20}O$
Molecular Weight: 192.30200
hept-1-yn-4-ol + 1-hexyne

Preparation of Alkynols and Brominated Alkynols, and Characterization Data.

1-heptyn-4-ol was prepared according to a literature procedure[1]: To a stirred solution of 200 mL dry THF, 17.40 g of 1,2-diiodoethane (62 mmol; 1.1 equiv), 9.0 mL propargyl bromide in 80% toluene (83 mmol, 1.5 equiv) , and 18.20 g of zinc dust (278 mmol, 5.0 equiv), 5.0 mL of butyraldehyde (56 mmol, 1.0 equiv) was added and allowed to stir for 5-10 minutes at room temperature. The solution was then sonicated at room temperature for 3h. The solution was then quenched with $NH_4Cl_{(aq)}$, extracted with ether (ca. 3 x 25mL), and dried over $MgSO_4$. Excess solvent was removed *in vacuo* and the dark brown product was purified over a silica column (8/2 hexanes:ethyl acetate) to give 2.25 g (36%) of product as a golden oil. ^1H-NMR ($CDCl_3$) δ: 0.9-1.0 (m, 3H), 1.2-1.6 (m, 4H), 2.0-2.1 (m, 1H), 2.32-2.42 (m, 2H), 3.65-3.85 (m, 2H); $^{13}C\{^1H\}$ NMR ($CDCl_3$) δ: 14.0, 18.8, 27.4, 38.4, 69.8, 70.7, 81.2.[2]

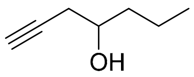

hept-1-yn-4-ol
Chemical Formula: $C_7H_{12}O$
Molecular Weight: 112.17200

[1] Lee, A. S.-Y.; Chu, S.-F.; Chang, Y.-T.; Wang, S.-H. Synthesis of Homopropargyl Alcohols via Sonochemical Barbier-Type Reaction. *Tetrahedron Lett.* **2004**, *45* (7), 1551–1553. https://doi.org/10.1016/j.tetlet.2003.12.058.

[2] Jõgi, A.; Mäeorg, U. Zn Mediated Regioselective Barbier Reaction of Propargylic Bromides in THF/aq. NH_4Cl Solution. *Molecules* **2001**, *6*, 964-968. https://doi.org/10.3390/61200964

1-bromohept-1-yn-4-ol was prepared using general procedure **B** to afford 907 mg (79 %) of product from 6.0 mmol of 1-heptyn-4-ol in 12 mL of dry acetone; NMR spectral data was consistent with literature values.[3]

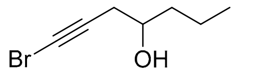

1-bromohept-1-yn-4-ol
Chemical Formula: $C_7H_{11}BrO$
Molecular Weight: 191.06800

6-Bromo-5-Hexyn-3-ol was prepared using general procedure **B** to afford 283 mg (53%) of product from 3.0 mmol of 5-hexyn-3-ol in 6 mL of dry acetone; yellow oil; R_f =0.43; ^{1}H NMR (400 MHz, $CDCl_3$): 0.97 (t, J = 7.6 Hz, 3H), 1.49-1.65 (m, 2H), 1.83 (d, J = 5.1 Hz, 1H), 2.36 (dd, J = 6.7 Hz, J = 16.8 Hz, 1H), 2.46 (dd, J = 4.9 Hz, J = 16.6 Hz, 1H), 3.65-3.73 (m, 1H). ^{13}C{^{1}H} NMR (100 MHz, $CDCl_3$): 9.8, 27.9, 29.0, 40.1, 71.1, 76.9.

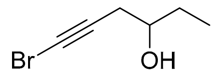

6-Bromo-5-Hexyn-3-ol
Chemical Formula: C_6H_9BrO
Molecular Weight: 177.04100

[3] Reddy, M. S.; Kumar, Y. K.; Thirupathi, N. A New Synthesis of γ-Butyrolactones via $AuCl_3$- or Hg(II)-Catalyzed Intramolecular Hydroalkoxylation of 4-Bromo-3-Yn-1-Ols. *Org. Lett.* **2012**, *14*, 824–827. https://doi.org/10.1021/ol2033493.

^{1}H and ^{13}C{^{1}H} APT NMR Spectra for Diynes 3-22, and Representative Spectra of a Brominated Alkynol.

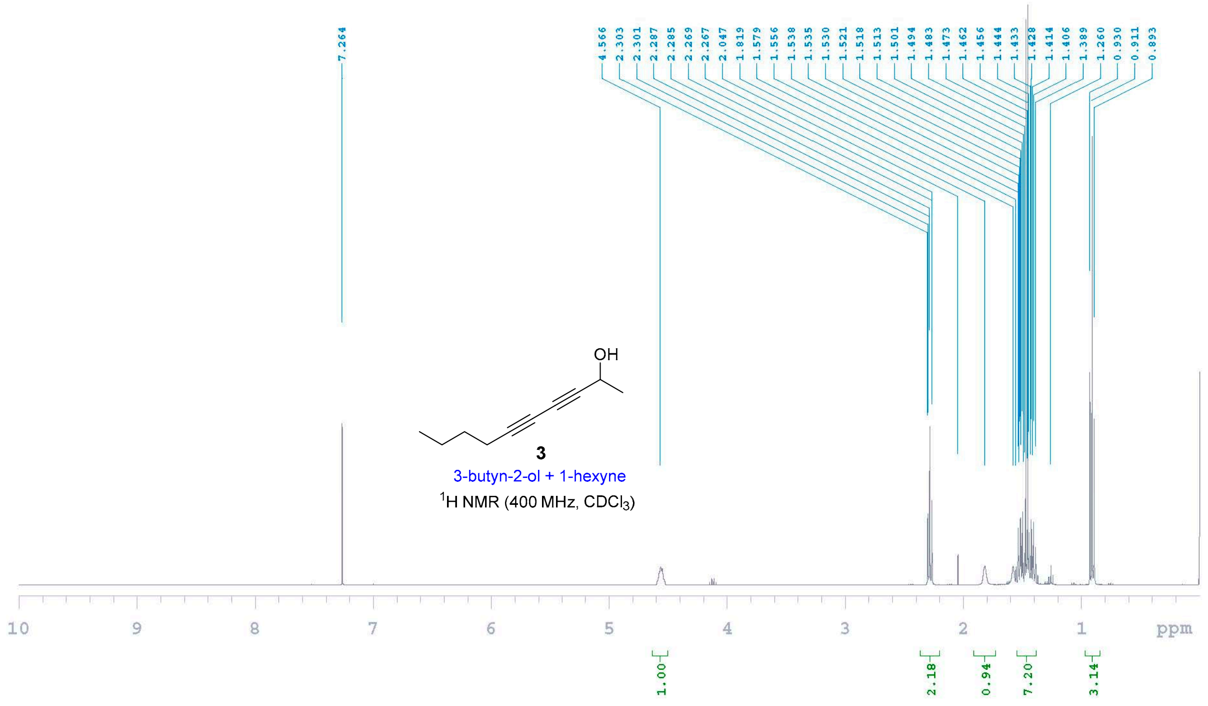

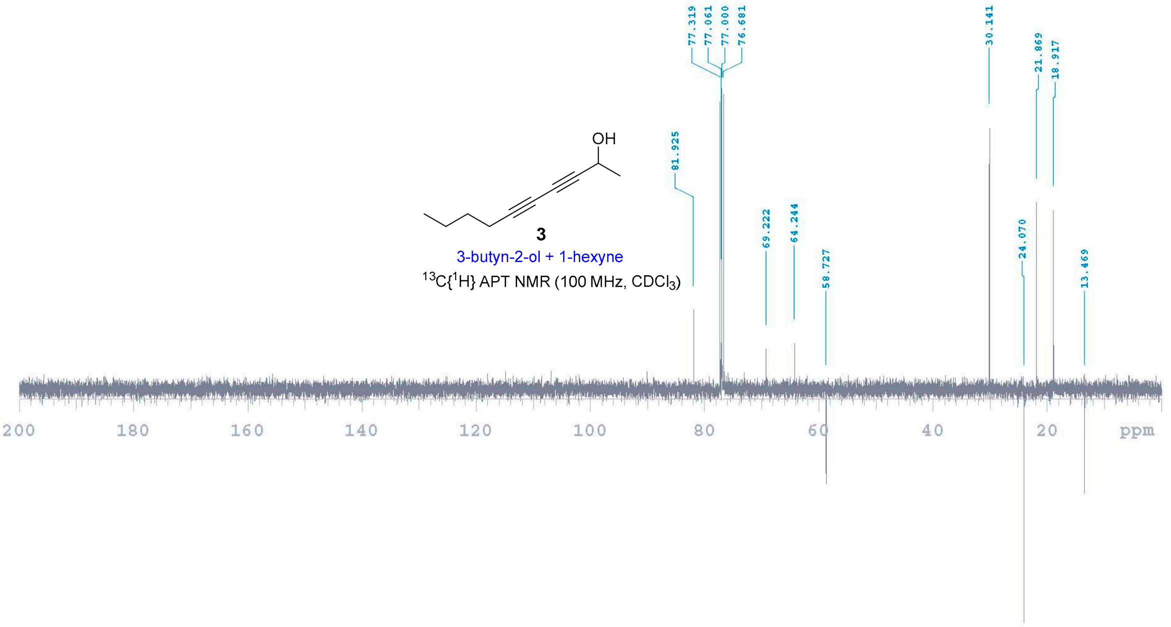

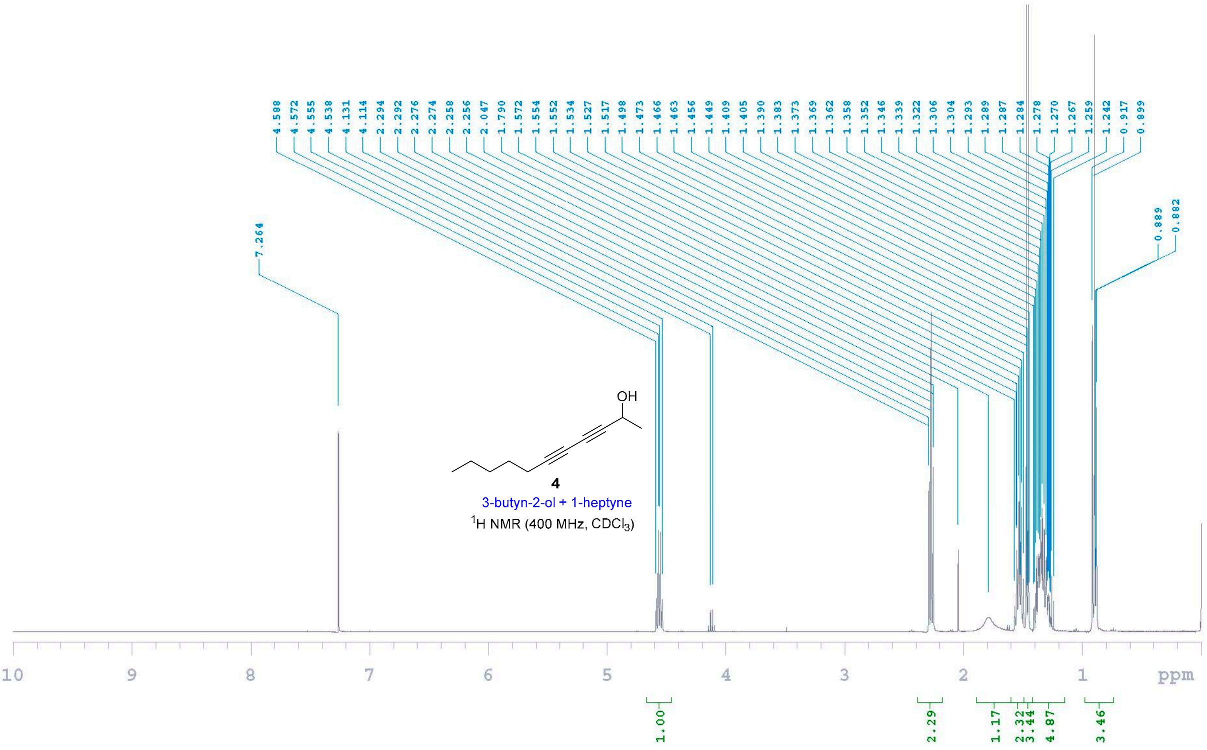
4
3-butyn-2-ol + 1-heptyne
^{1}H NMR (400 MHz, CDCl$_3$)
7.264
4.588 4.572 4.555 4.538 4.131 4.114 2.294 2.292 2.276 2.274 2.258 2.256 2.047 1.790 1.572 1.554 1.552 1.534 1.527 1.517 1.498 1.473 1.466 1.463 1.456 1.449 1.409 1.405 1.390 1.383 1.373 1.369 1.362 1.358 1.352 1.346 1.339 1.322 1.306 1.304 1.293 1.289 1.287 1.284 1.278 1.270 1.267 1.259 1.242 0.917 0.899
0.889 0.882
1.00 2.29 1.17 2.32 3.44 4.87 3.46
10 9 8 7 6 5 4 3 2 1 ppm

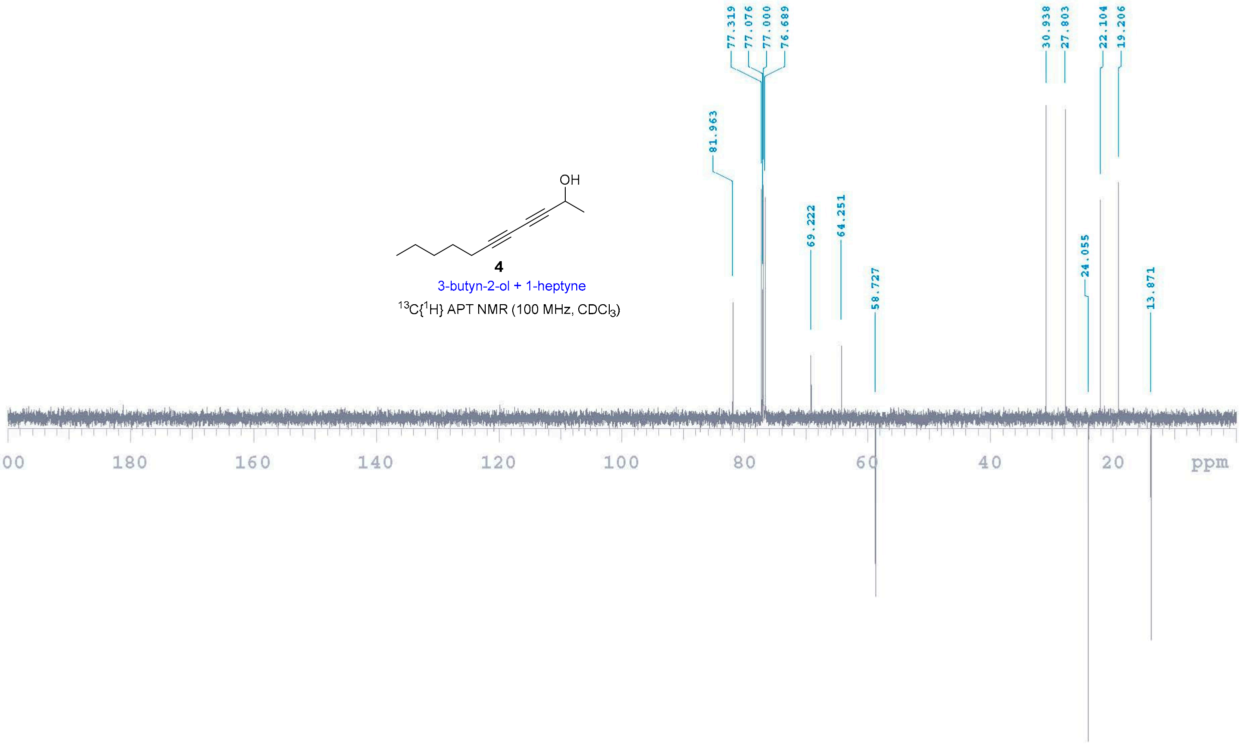
4
3-butyn-2-ol + 1-heptyne
^{13}C{^{1}H} APT NMR (100 MHz, CDCl$_3$)
77.319 77.076 77.000 76.689
81.963
69.222 64.251 58.727
30.938 27.803 22.104 19.206
24.055 13.871
200 180 160 140 120 100 80 60 40 20 ppm

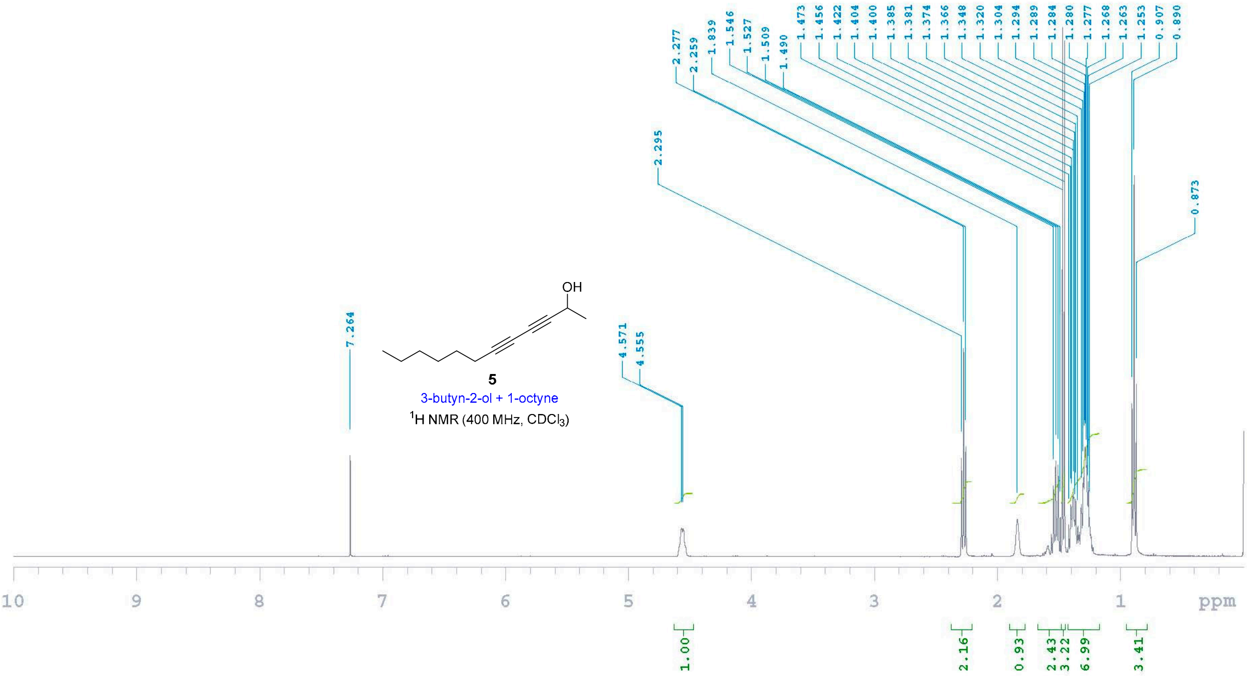
OH
5
3-butyn-2-ol + 1-octyne
1H NMR (400 MHz, CDCl3)
7.264
4.571
4.555
2.295
2.277
2.259
1.839
1.546
1.527
1.509
1.490
1.473
1.456
1.422
1.404
1.400
1.385
1.381
1.374
1.366
1.348
1.320
1.304
1.294
1.289
1.284
1.280
1.277
1.268
1.263
1.253
0.907
0.890
0.873
1.00
2.16
0.93
2.43
3.22
6.99
3.41
10
9
8
7
6
5
4
3
2
1
ppm

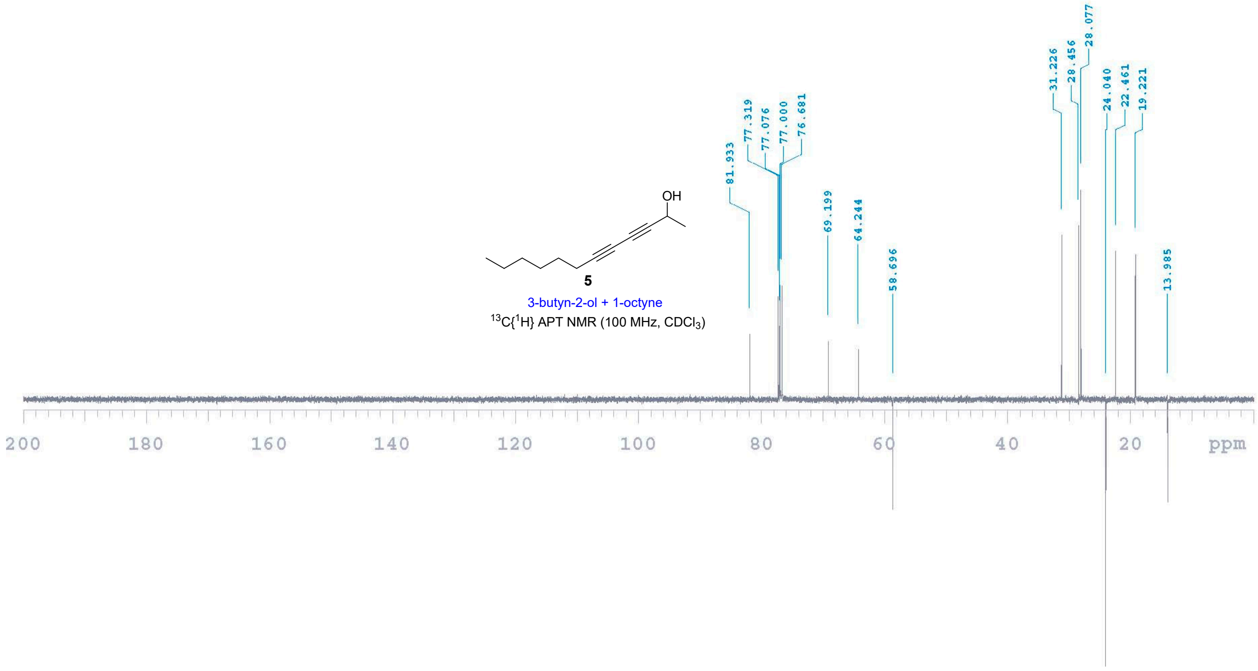
OH
5
3-butyn-2-ol + 1-octyne
13C{1H} APT NMR (100 MHz, CDCl3)
81.933
77.319
77.076
77.000
76.681
69.199
64.244
58.696
31.226
28.456
28.077
24.040
22.461
19.221
13.985
200
180
160
140
120
100
80
60
40
20
ppm

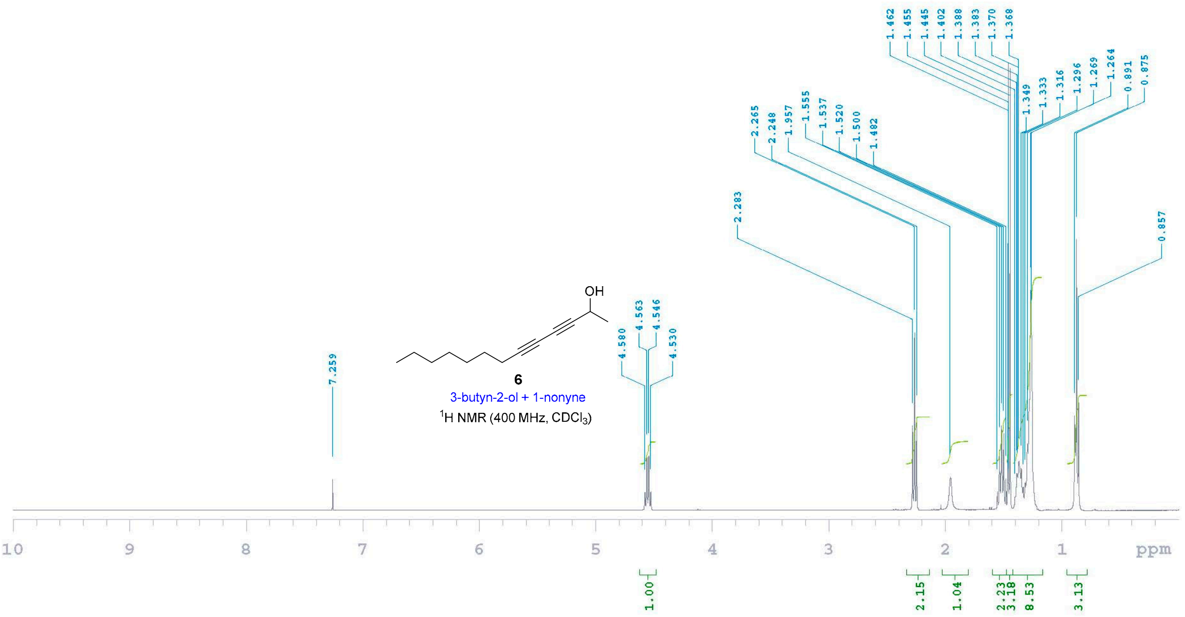
OH
6
3-butyn-2-ol + 1-nonyne
^{1}H NMR (400 MHz, CDCl$_3$)
7.259
4.580 4.563 4.546 4.530
2.283 2.265 2.248 1.957
1.555 1.537 1.520 1.500 1.482
1.462 1.455 1.445 1.402 1.388 1.383 1.370 1.368 1.349 1.333 1.316 1.296 1.269 1.264
0.891 0.875 0.857
10 9 8 7 6 5 4 3 2 1 ppm
1.00 2.15 1.04 2.23 3.18 8.53 3.13

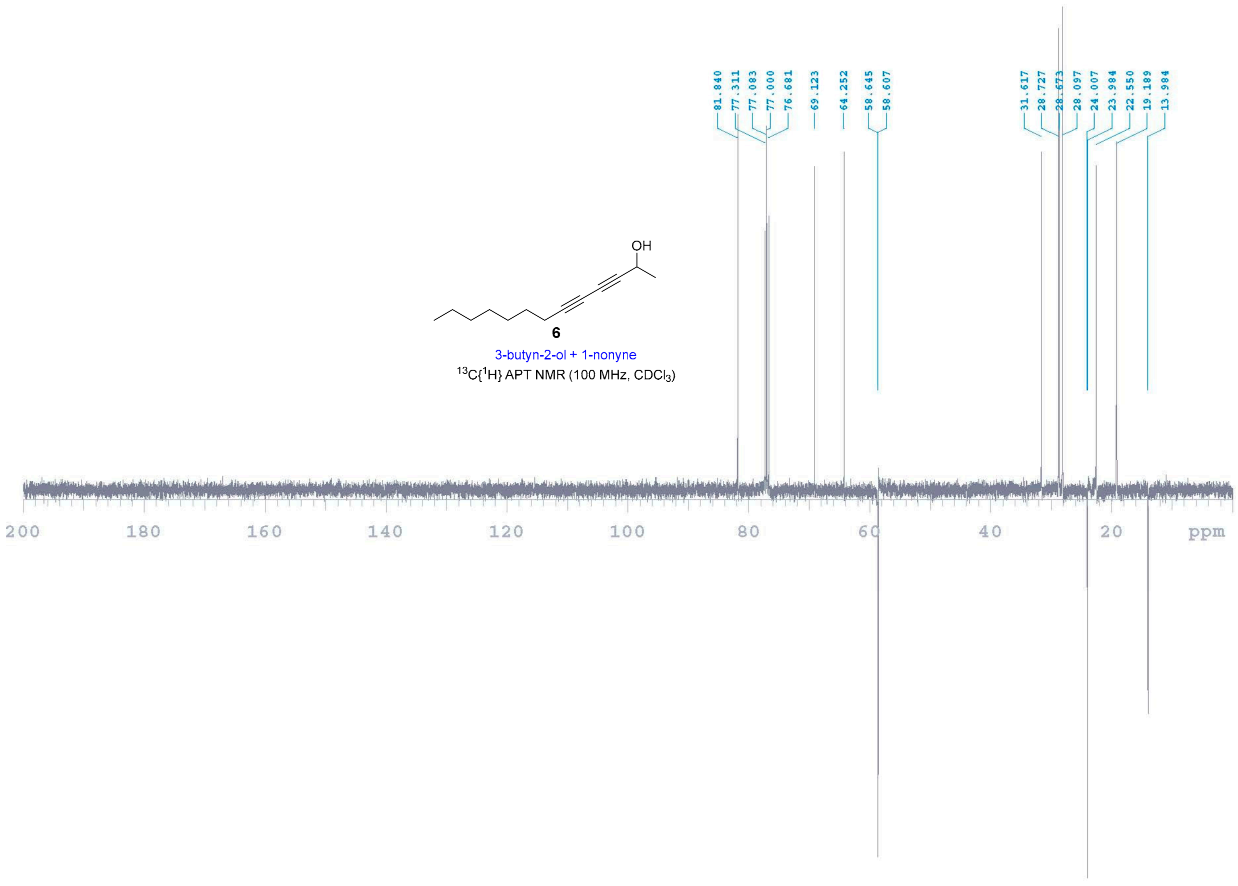
81.840 77.311 77.083 77.000 76.681 69.123 64.252 58.645 58.607
31.617 28.727 28.673 28.097 24.007 23.984 22.550 19.189 13.984
OH
6
3-butyn-2-ol + 1-nonyne
^{13}C{^{1}H} APT NMR (100 MHz, CDCl$_3$)
200 180 160 140 120 100 80 60 40 20 ppm

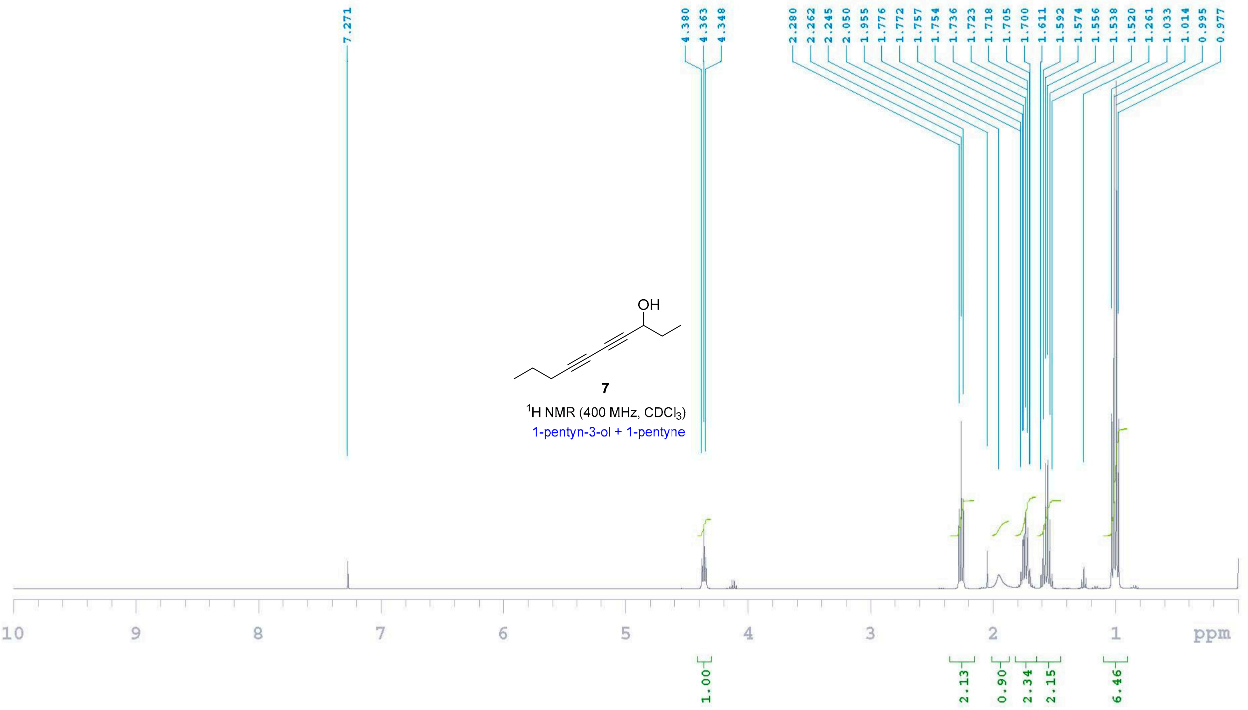
OH
7
1H NMR (400 MHz, CDCl3)
1-pentyn-3-ol + 1-pentyne
7.271
4.380 4.363 4.348
2.280 2.262 2.245 2.050 1.955 1.776 1.772 1.757 1.754 1.736 1.723 1.718 1.705 1.700 1.611 1.592 1.574 1.556 1.538 1.520 1.261 1.033 1.014 0.995 0.977
10 9 8 7 6 5 4 3 2 1 ppm
1.00 2.13 0.90 2.34 2.15 6.46

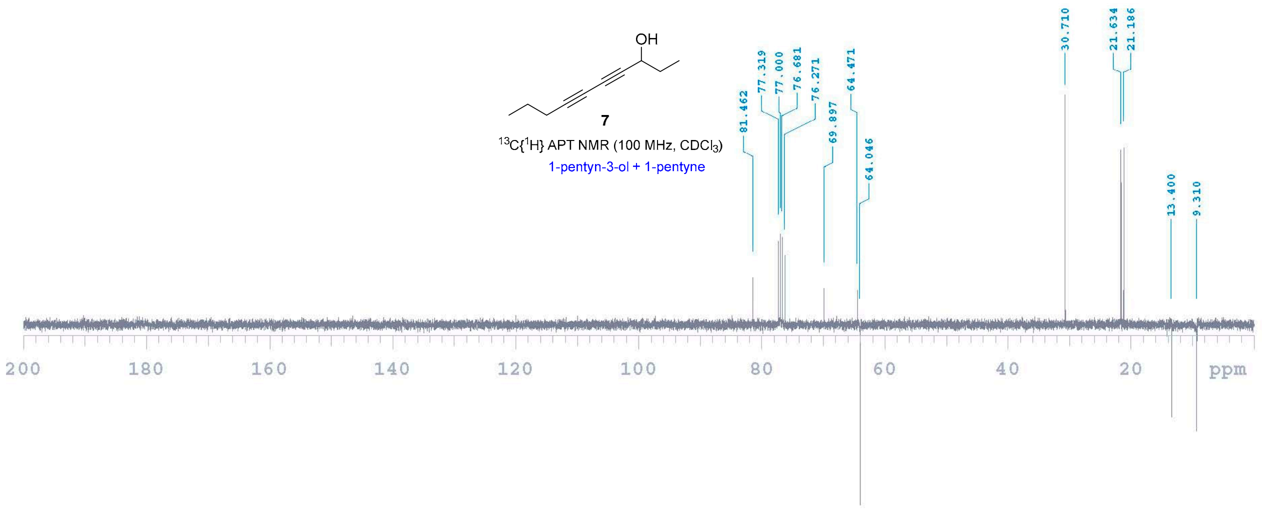
OH
7
13C{1H} APT NMR (100 MHz, CDCl3)
1-pentyn-3-ol + 1-pentyne
81.462 77.319 77.000 76.681 76.271 69.897 64.471 64.046 30.710 21.634 21.186 13.400 9.310
200 180 160 140 120 100 80 60 40 20 ppm

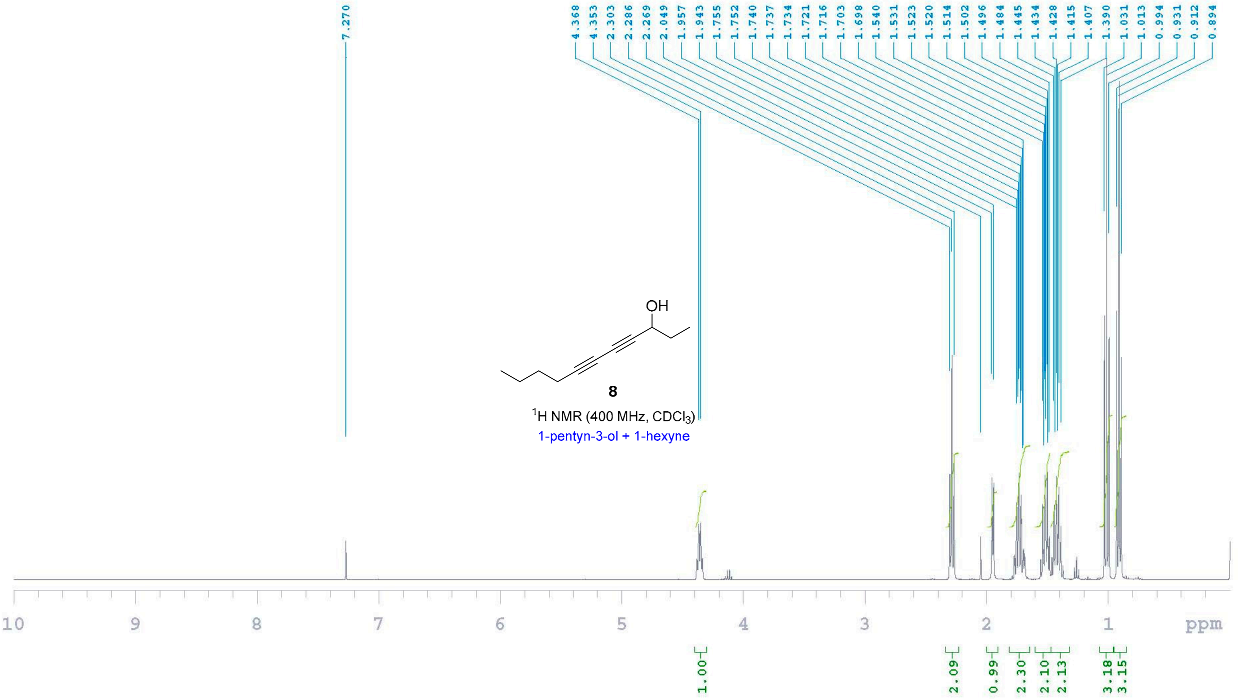
7.270
4.368 4.353 2.303 2.286 2.269 2.049 1.957 1.943 1.755 1.752 1.740 1.737 1.734 1.721 1.716 1.703 1.698 1.540 1.531 1.523 1.520 1.514 1.502 1.496 1.484 1.445 1.434 1.428 1.415 1.407 1.390 1.031 1.013 0.994 0.931 0.912 0.894
OH
8
1H NMR (400 MHz, CDCl3)
1-pentyn-3-ol + 1-hexyne
10 9 8 7 6 5 4 3 2 1 ppm
1.00 2.09 0.99 2.30 2.10 2.13 3.18 3.15

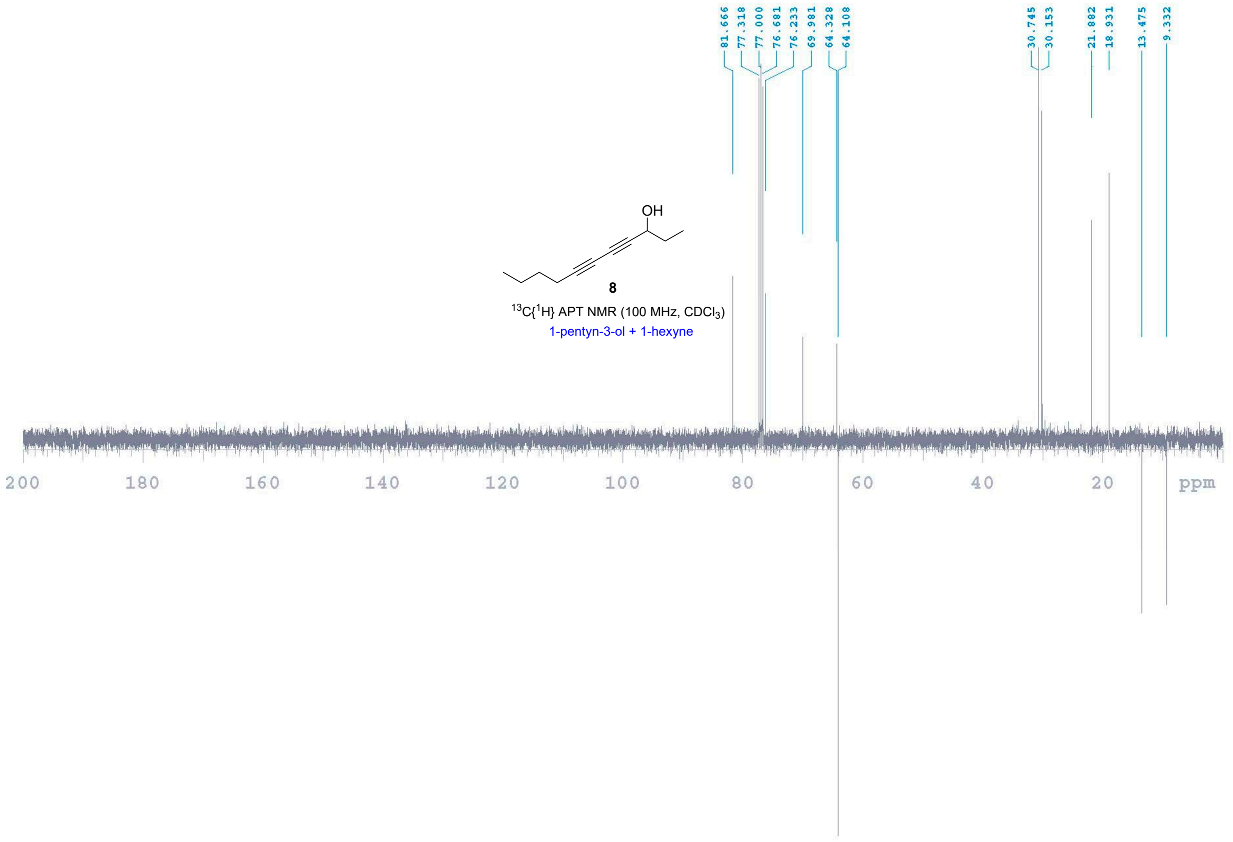
81.666 77.318 77.000 76.681 76.233 69.981 64.328 64.108 30.745 30.153 21.882 18.931 13.475 9.332
OH
8
13C{1H} APT NMR (100 MHz, CDCl3)
1-pentyn-3-ol + 1-hexyne
200 180 160 140 120 100 80 60 40 20 ppm

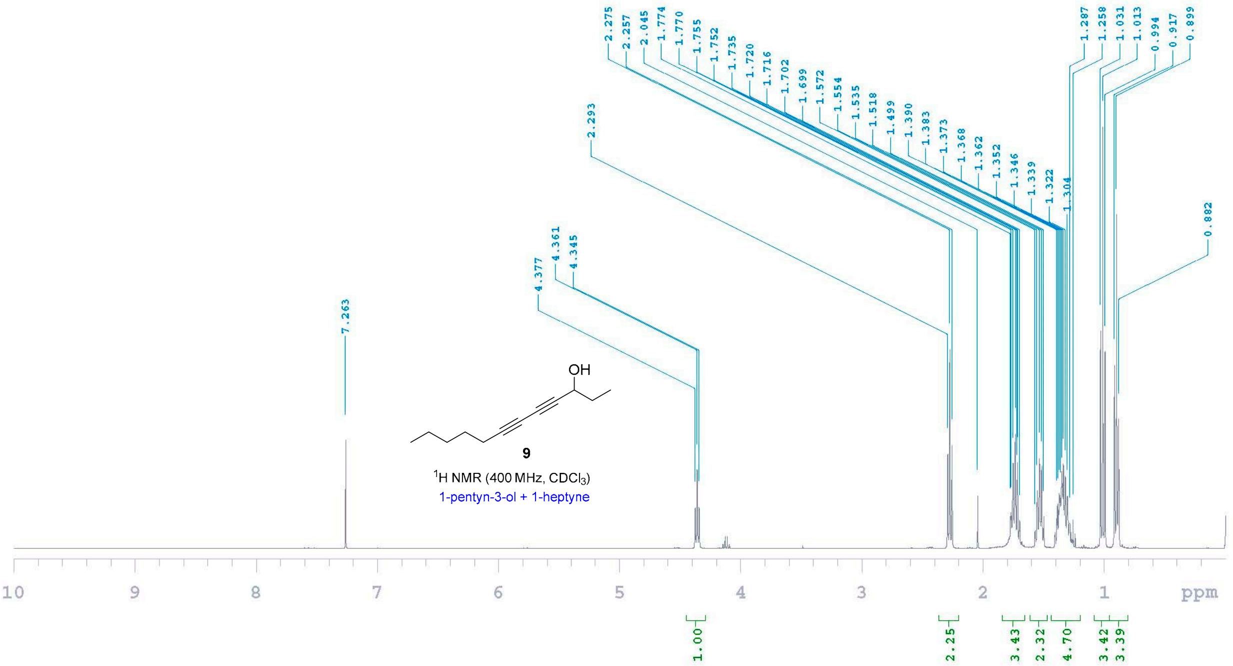
OH
9
^{1}H NMR (400 MHz, CDCl$_3$)
1-pentyn-3-ol + 1-heptyne
7.263
4.377 4.361 4.345
2.293 2.275 2.257 2.045 1.774 1.770 1.755 1.752 1.735 1.720 1.716 1.702 1.699 1.572 1.554 1.535 1.518 1.499 1.390 1.383 1.373 1.368 1.362 1.352 1.346 1.339 1.322 1.304 1.287 1.258 1.031 1.013 0.994 0.917 0.899 0.882
1.00 2.25 3.43 2.32 4.70 3.42 3.39
10 9 8 7 6 5 4 3 2 1 ppm

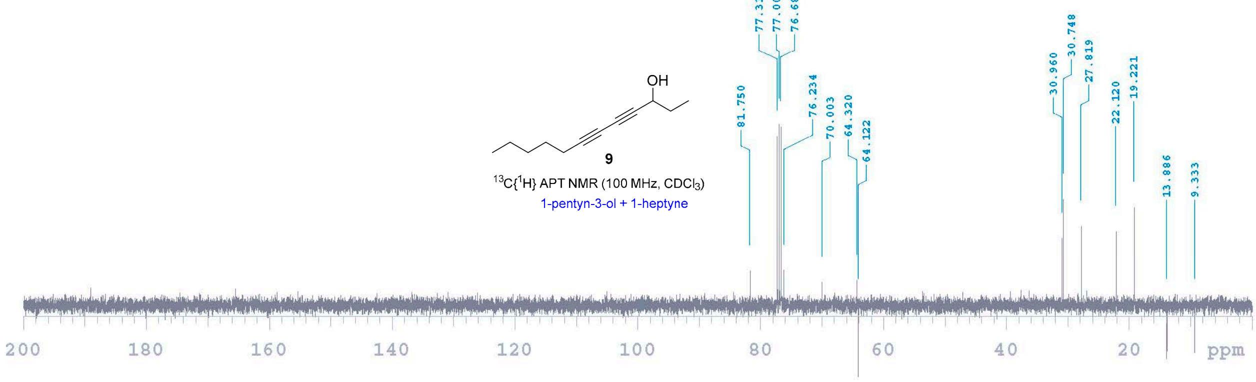
OH
9
^{13}C{^{1}H} APT NMR (100 MHz, CDCl$_3$)
1-pentyn-3-ol + 1-heptyne
81.750 77.319 77.000 76.681 76.234 70.003 64.320 64.122
30.960 30.748 27.819 22.120 19.221 13.886 9.333
200 180 160 140 120 100 80 60 40 20 ppm

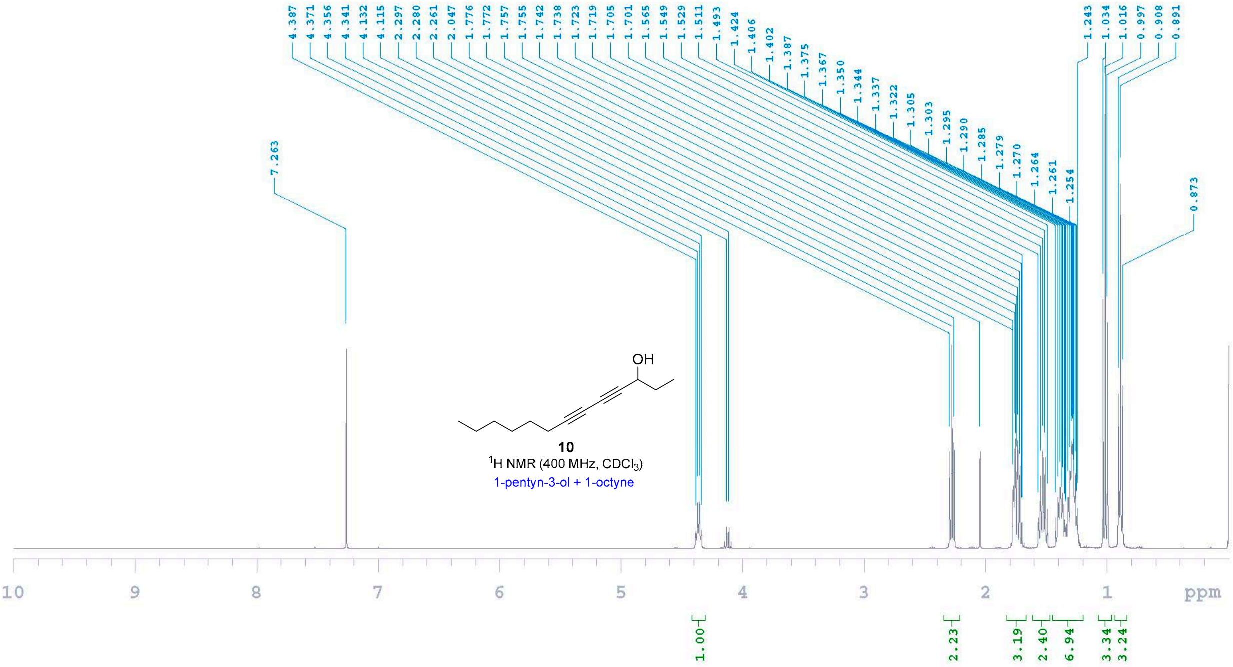

10

^{1}H NMR (400 MHz, $CDCl_3$)

1-pentyn-3-ol + 1-octyne

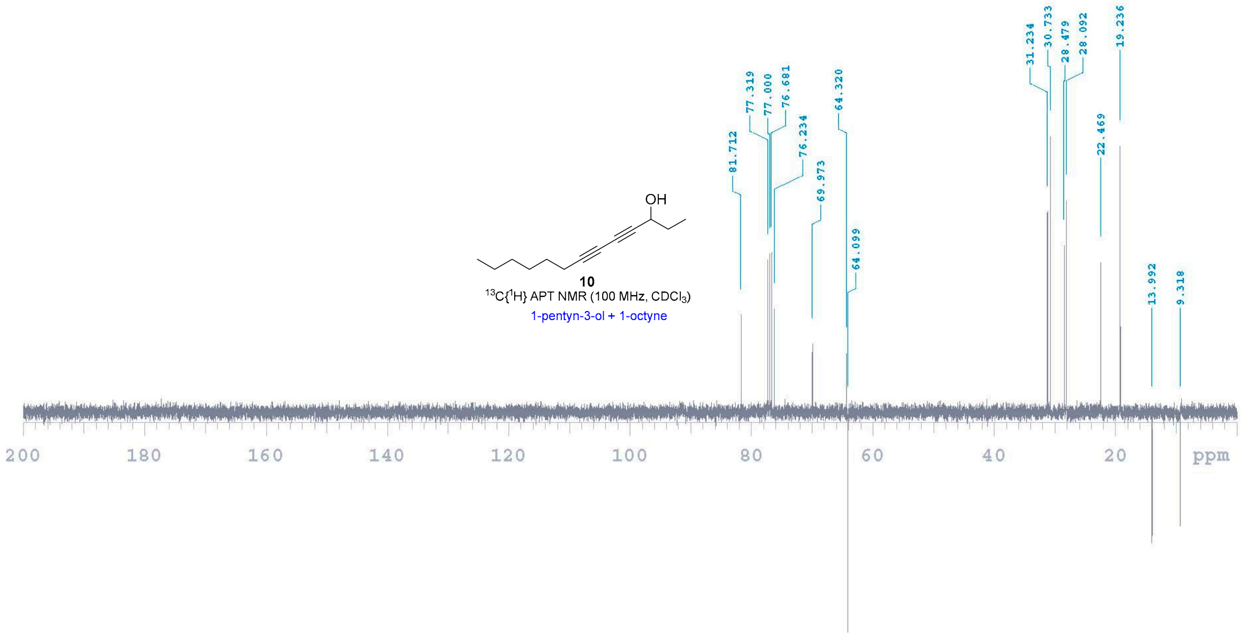

10

$^{13}C\{^1H\}$ APT NMR (100 MHz, $CDCl_3$)

1-pentyn-3-ol + 1-octyne

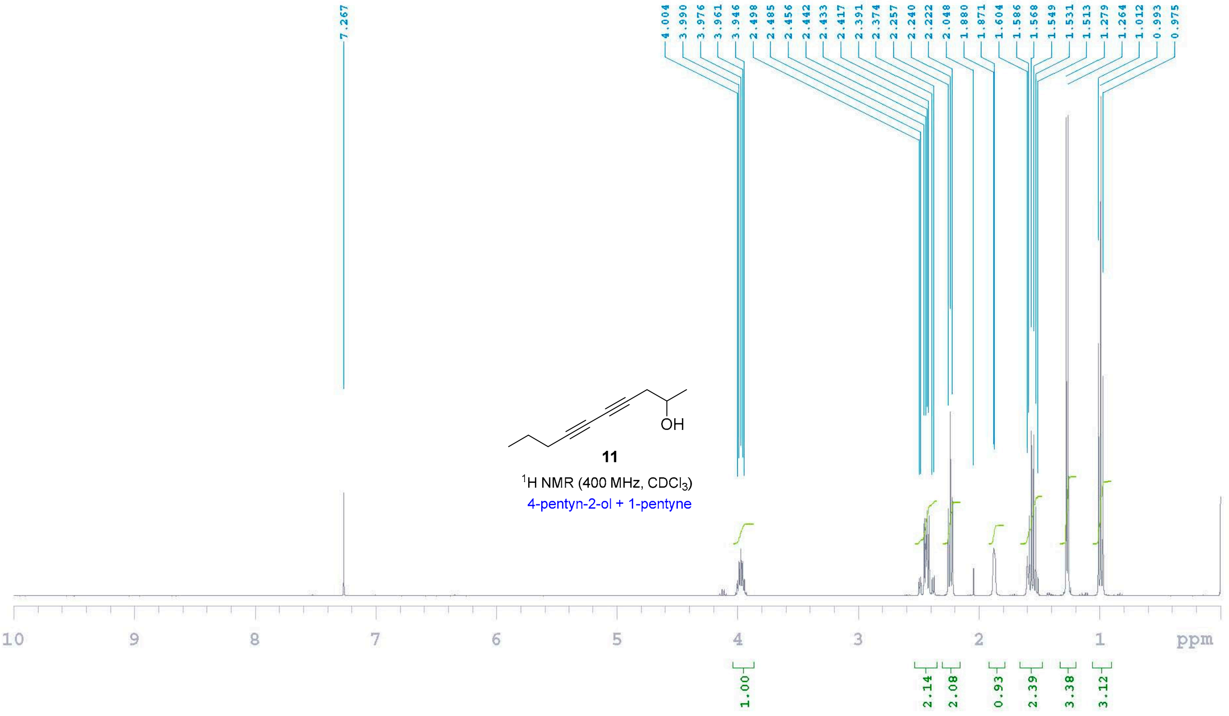
7.267
4.004
3.990
3.976
3.961
3.946
2.498
2.485
2.456
2.442
2.433
2.417
2.391
2.374
2.257
2.240
2.222
2.048
1.880
1.871
1.604
1.586
1.568
1.549
1.531
1.513
1.279
1.264
1.012
0.993
0.975
OH
11
^{1}H NMR (400 MHz, $CDCl_3$)
4-pentyn-2-ol + 1-pentyne
10
9
8
7
6
5
4
3
2
1
ppm
1.00
2.14
2.08
0.93
2.39
3.38
3.12

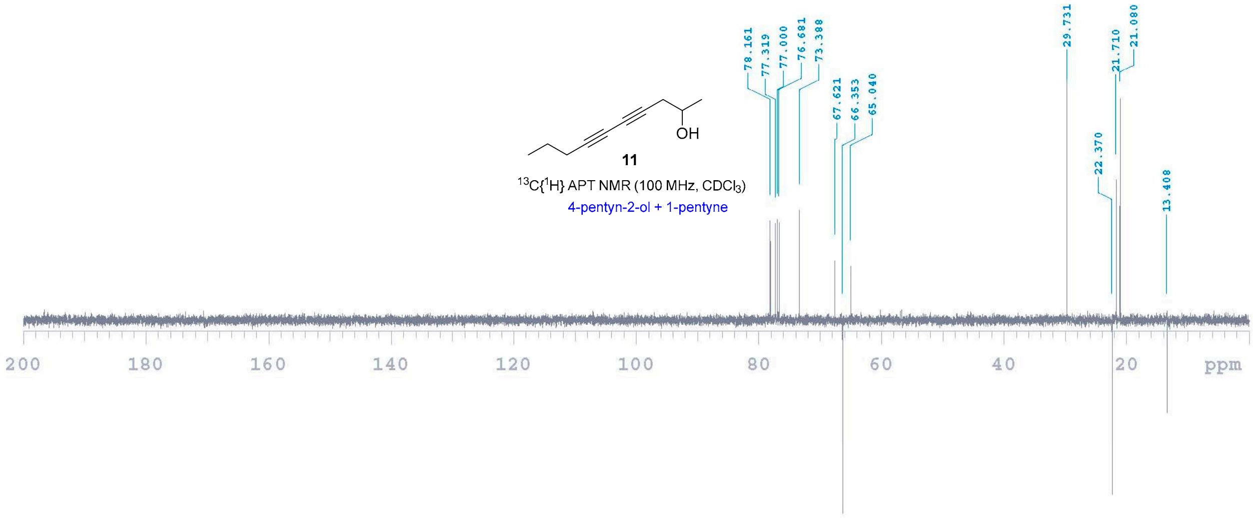
78.161
77.319
77.000
76.681
73.388
67.621
66.353
65.040
29.731
21.710
21.080
22.370
13.408
OH
11
$^{13}C\{^1H\}$ APT NMR (100 MHz, $CDCl_3$)
4-pentyn-2-ol + 1-pentyne
200
180
160
140
120
100
80
60
40
20
ppm

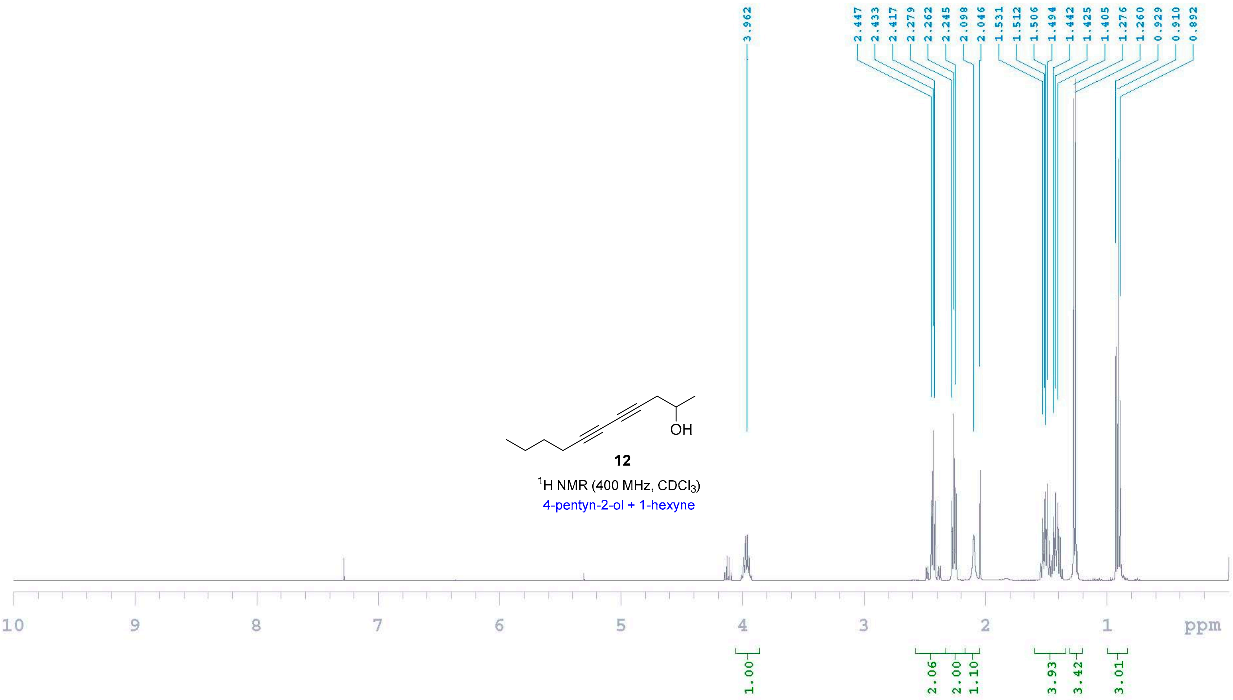
OH
12
1H NMR (400 MHz, CDCl3)
4-pentyn-2-ol + 1-hexyne
3.962
2.447 2.433 2.417 2.279 2.262 2.245 2.098 2.046 1.531 1.512 1.506 1.494 1.442 1.425 1.405 1.276 1.260 0.929 0.910 0.892
10 9 8 7 6 5 4 3 2 1 ppm
1.00 2.06 2.00 1.10 3.93 3.42 3.01

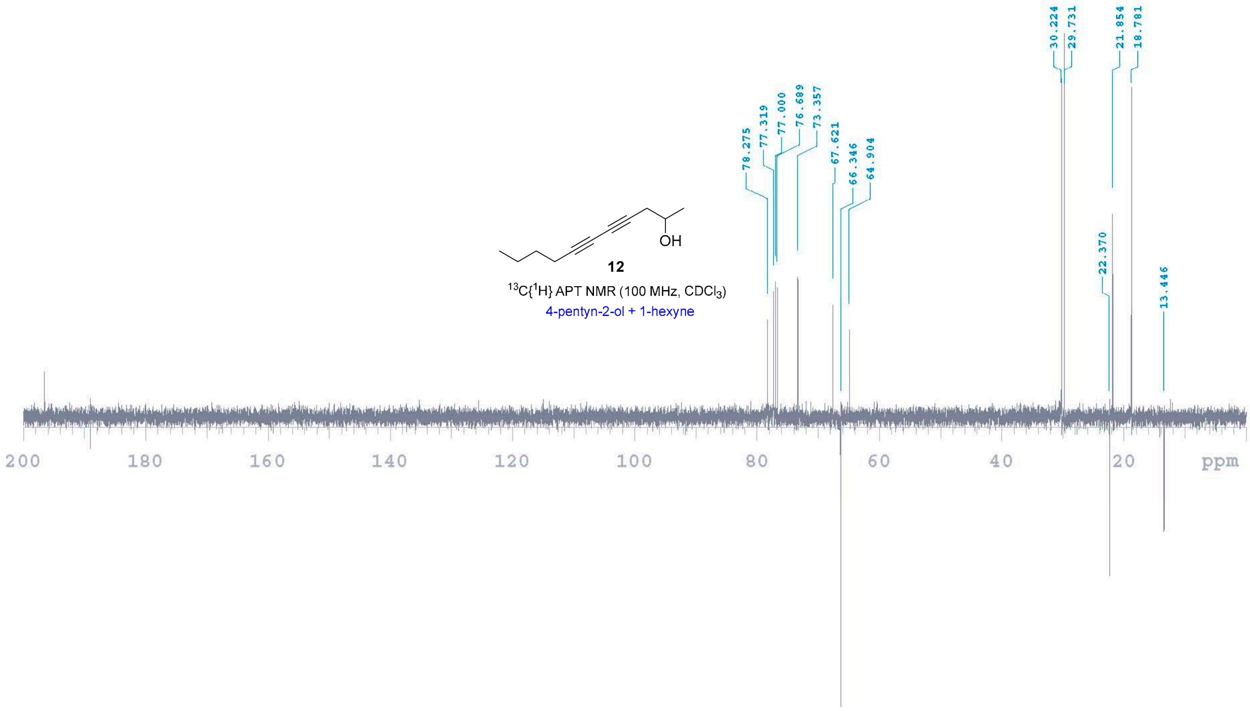
OH
12
13C{1H} APT NMR (100 MHz, CDCl3)
4-pentyn-2-ol + 1-hexyne
78.275 77.319 77.000 76.689 73.357 67.621 66.346 64.904
30.224 29.731 21.854 18.781
22.370
13.446
200 180 160 140 120 100 80 60 40 20 ppm

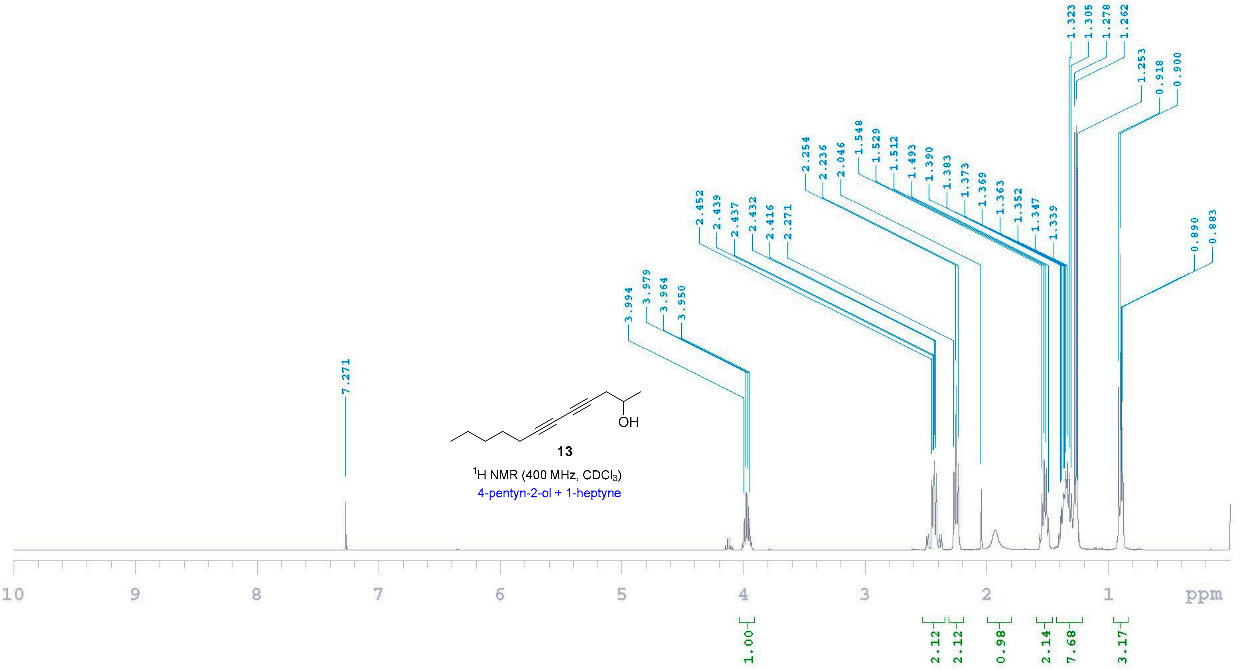
13
^{1}H NMR (400 MHz, CDCl$_3$)
4-pentyn-2-ol + 1-heptyne
OH
7.271
3.994 3.979 3.964 3.950
2.452 2.439 2.437 2.432 2.416 2.271
2.254 2.236 2.046
1.548 1.529 1.512 1.493 1.390 1.383 1.373 1.369 1.363 1.352 1.347 1.339
1.323 1.305 1.278 1.262 1.253 0.918 0.900 0.890 0.883
1.00 2.12 2.12 0.98 2.14 7.68 3.17
10 9 8 7 6 5 4 3 2 1 ppm

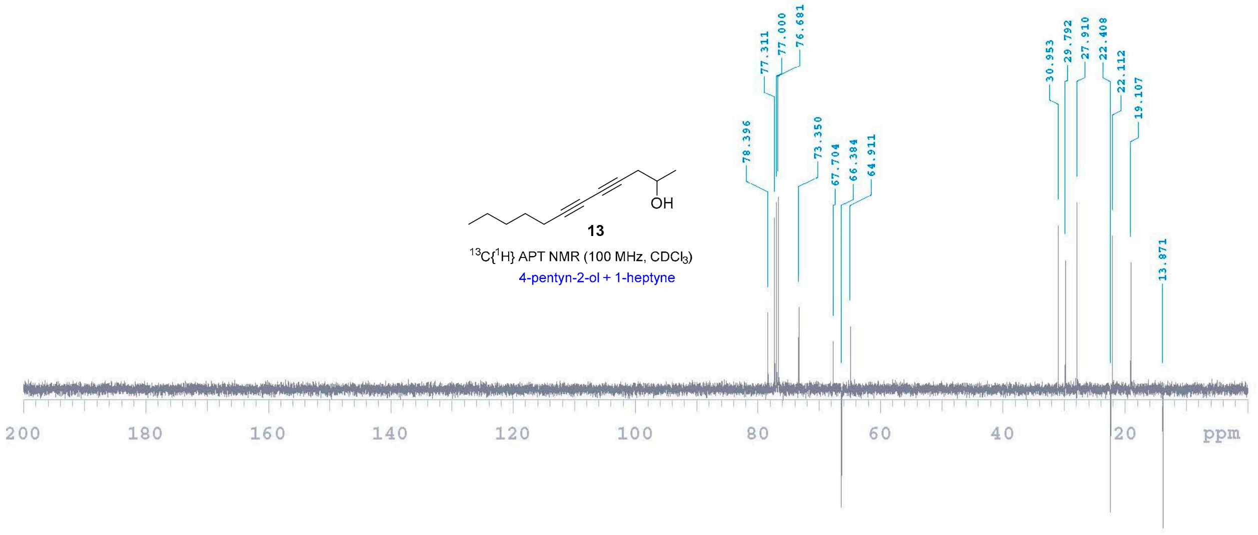
13
^{13}C{^{1}H} APT NMR (100 MHz, CDCl$_3$)
4-pentyn-2-ol + 1-heptyne
OH
78.396 77.311 77.000 76.681 73.350 67.704 66.384 64.911
30.953 29.792 27.910 22.408 22.112 19.107 13.871
200 180 160 140 120 100 80 60 40 20 ppm

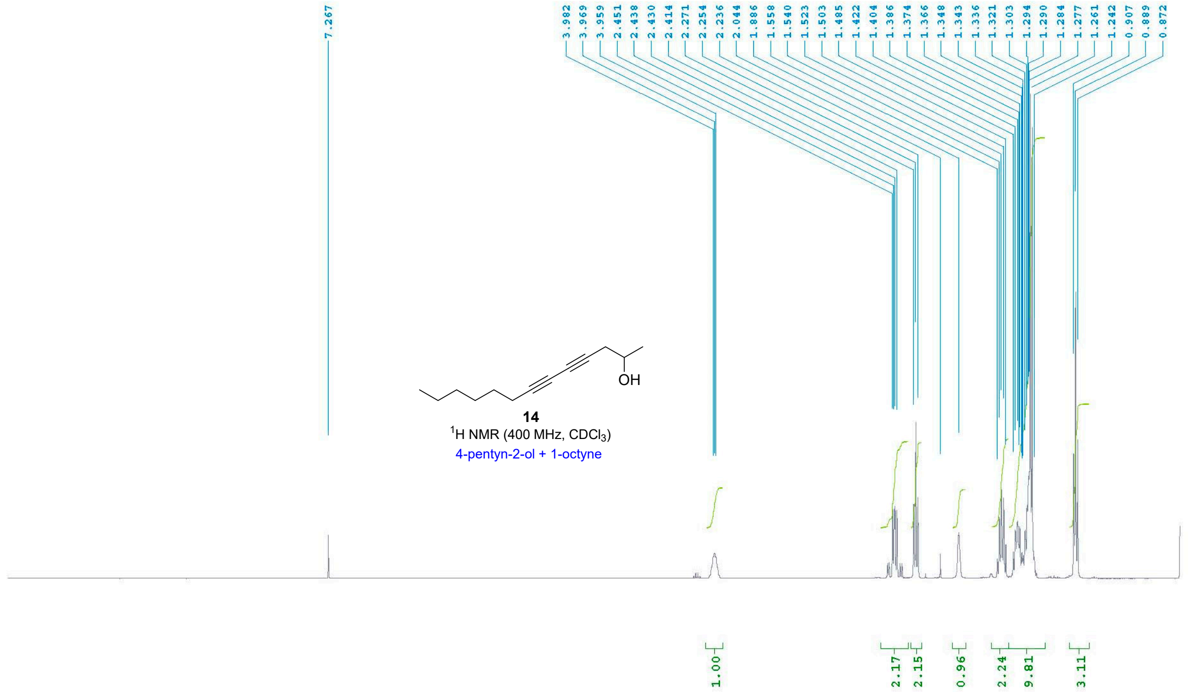
7.267
3.982
3.969
3.959
2.451
2.438
2.430
2.414
2.271
2.254
2.236
2.044
1.886
1.558
1.540
1.523
1.503
1.485
1.422
1.404
1.386
1.374
1.366
1.348
1.343
1.336
1.321
1.303
1.294
1.290
1.284
1.277
1.261
1.242
0.907
0.889
0.872
OH
14
^{1}H NMR (400 MHz, CDCl$_3$)
4-pentyn-2-ol + 1-octyne
1.00
2.17
2.15
0.96
2.24
9.81
3.11

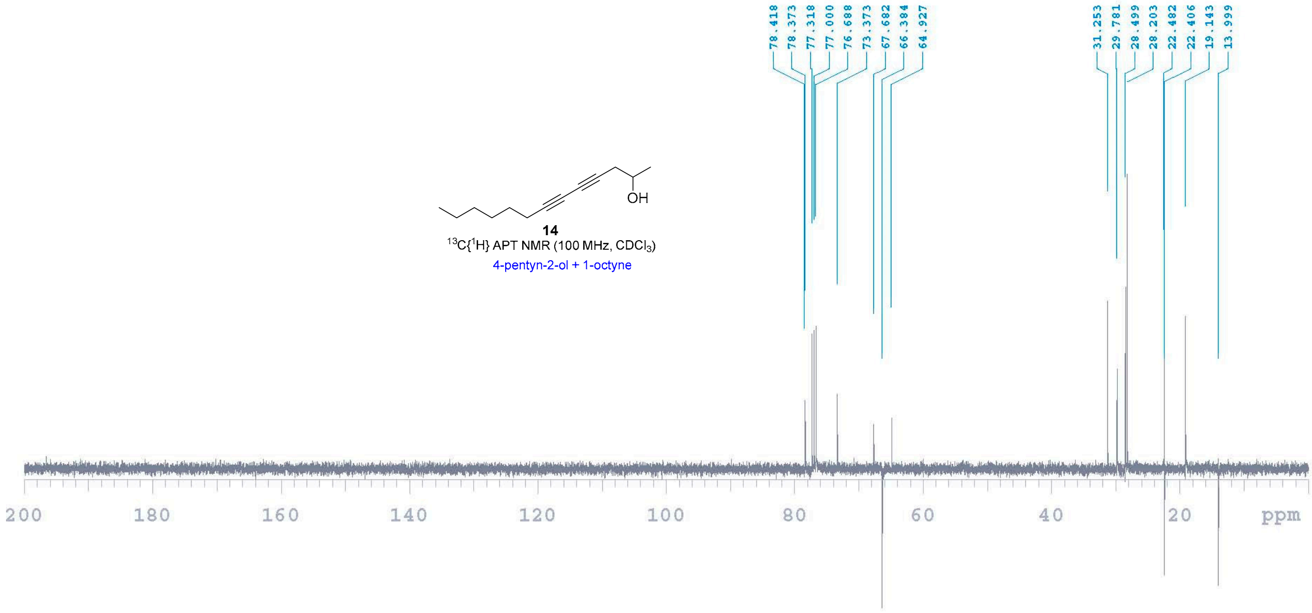
78.418
78.373
77.318
77.000
76.688
73.373
67.682
66.384
64.927
31.253
29.781
28.499
28.203
22.482
22.406
19.143
13.999
OH
14
^{13}C{^{1}H} APT NMR (100 MHz, CDCl$_3$)
4-pentyn-2-ol + 1-octyne
200
180
160
140
120
100
80
60
40
20
ppm

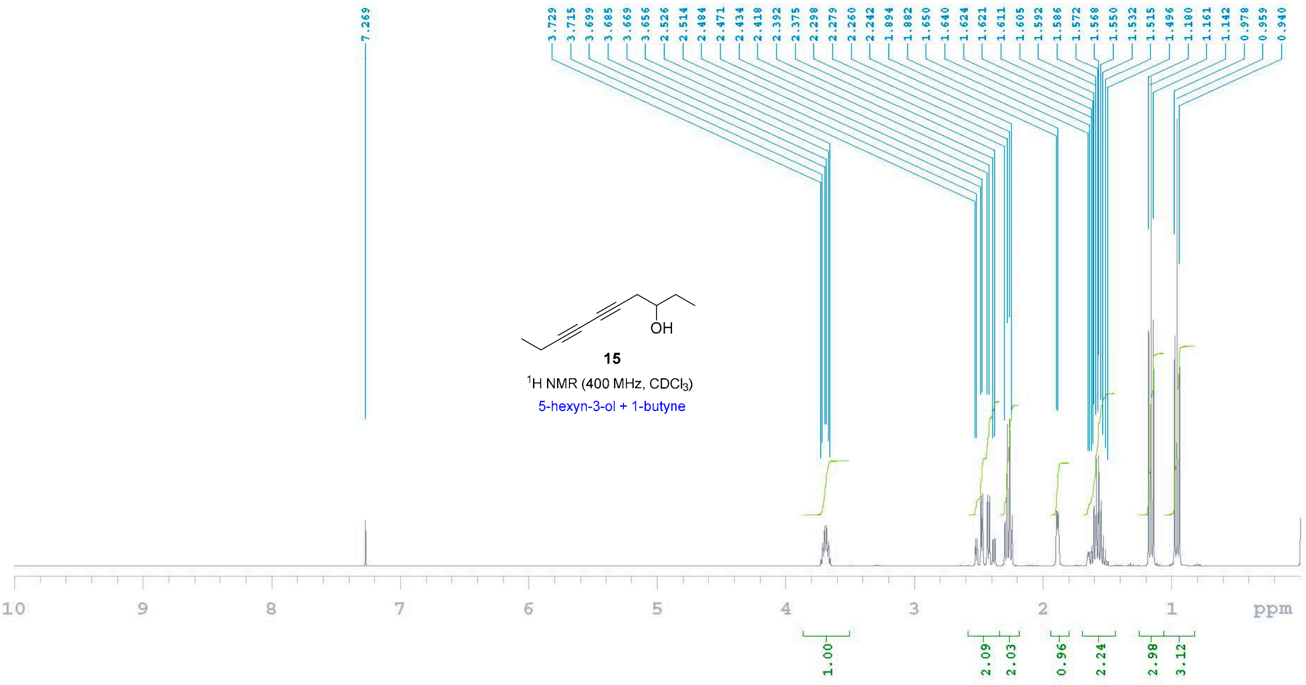
7.269
3.729 3.715 3.699 3.685 3.669 3.656 2.526 2.514 2.484 2.471 2.434 2.418 2.392 2.375 2.298 2.279 2.260 2.242 1.894 1.882 1.650 1.640 1.624 1.621 1.611 1.605 1.592 1.586 1.572 1.568 1.550 1.532 1.515 1.496 1.180 1.161 1.142 0.978 0.959 0.940
OH
15
1H NMR (400 MHz, CDCl3)
5-hexyn-3-ol + 1-butyne
10 9 8 7 6 5 4 3 2 1 ppm
1.00 2.09 2.03 0.96 2.24 2.98 3.12

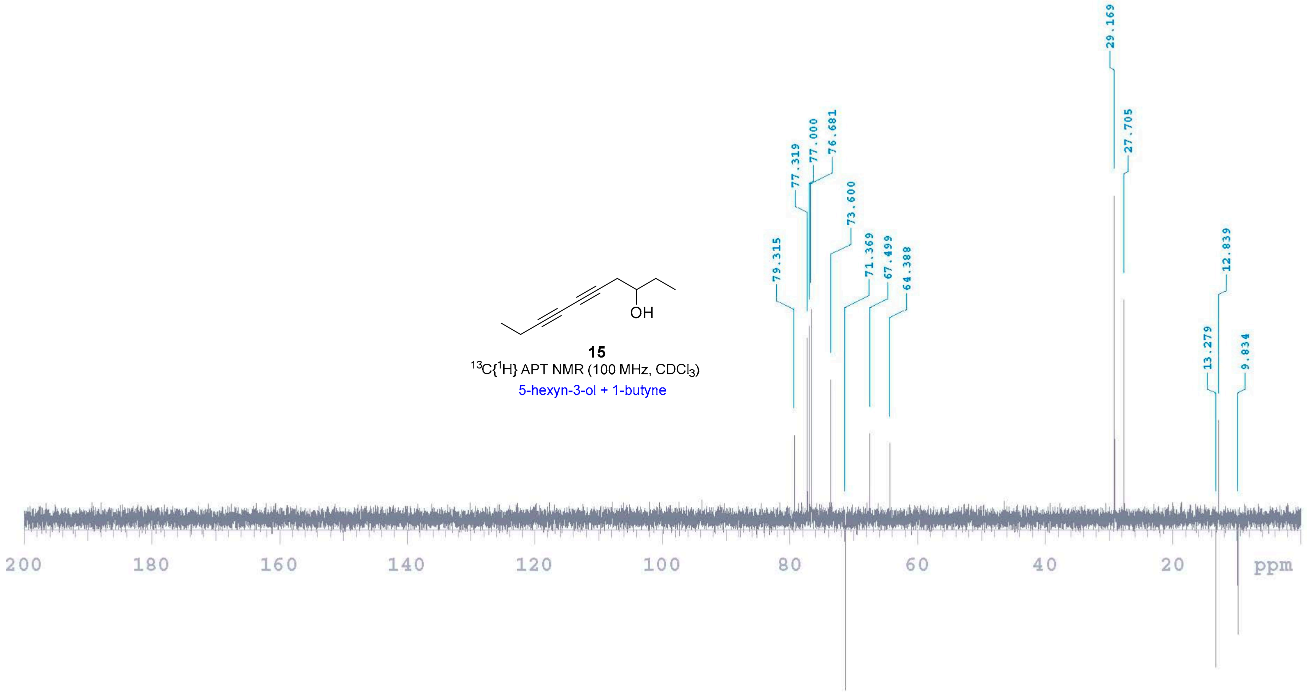
29.169
27.705
77.319 77.000 76.681
73.600
79.315
71.369 67.499 64.388
12.839
13.279
9.834
OH
15
13C{1H} APT NMR (100 MHz, CDCl3)
5-hexyn-3-ol + 1-butyne
200 180 160 140 120 100 80 60 40 20 ppm

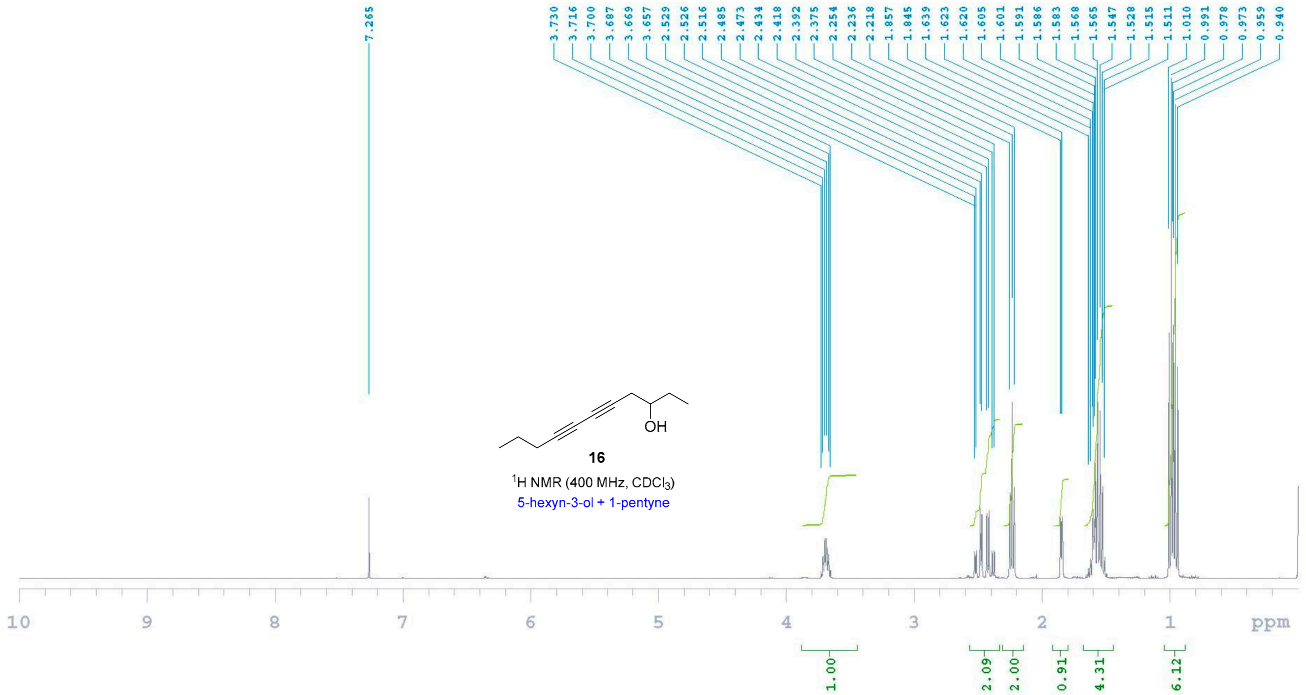
7.265
3.730 3.716 3.700 3.687 3.669 3.657 2.529 2.526 2.516 2.485 2.473 2.434 2.418 2.392 2.375 2.254 2.236 2.218 1.857 1.845 1.639 1.623 1.620 1.605 1.601 1.591 1.586 1.583 1.568 1.565 1.547 1.528 1.515 1.511 1.010 0.991 0.978 0.973 0.959 0.940
OH
16
^{1}H NMR (400 MHz, $CDCl_3$)
5-hexyn-3-ol + 1-pentyne
10 9 8 7 6 5 4 3 2 1 ppm
1.00 2.09 2.00 0.91 4.31 6.12

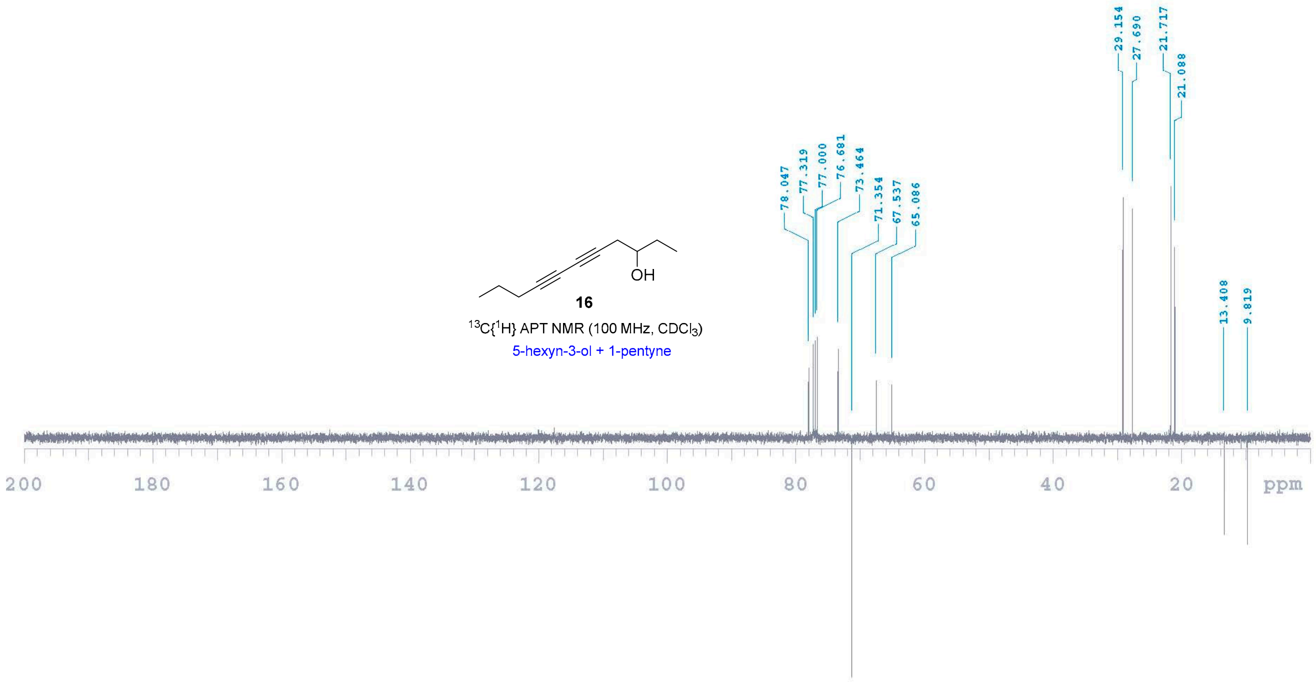
29.154 27.690 21.717 21.088
78.047 77.319 77.000 76.681 73.464 71.354 67.537 65.086
13.408 9.819
OH
16
$^{13}C\{^1H\}$ APT NMR (100 MHz, $CDCl_3$)
5-hexyn-3-ol + 1-pentyne
200 180 160 140 120 100 80 60 40 20 ppm

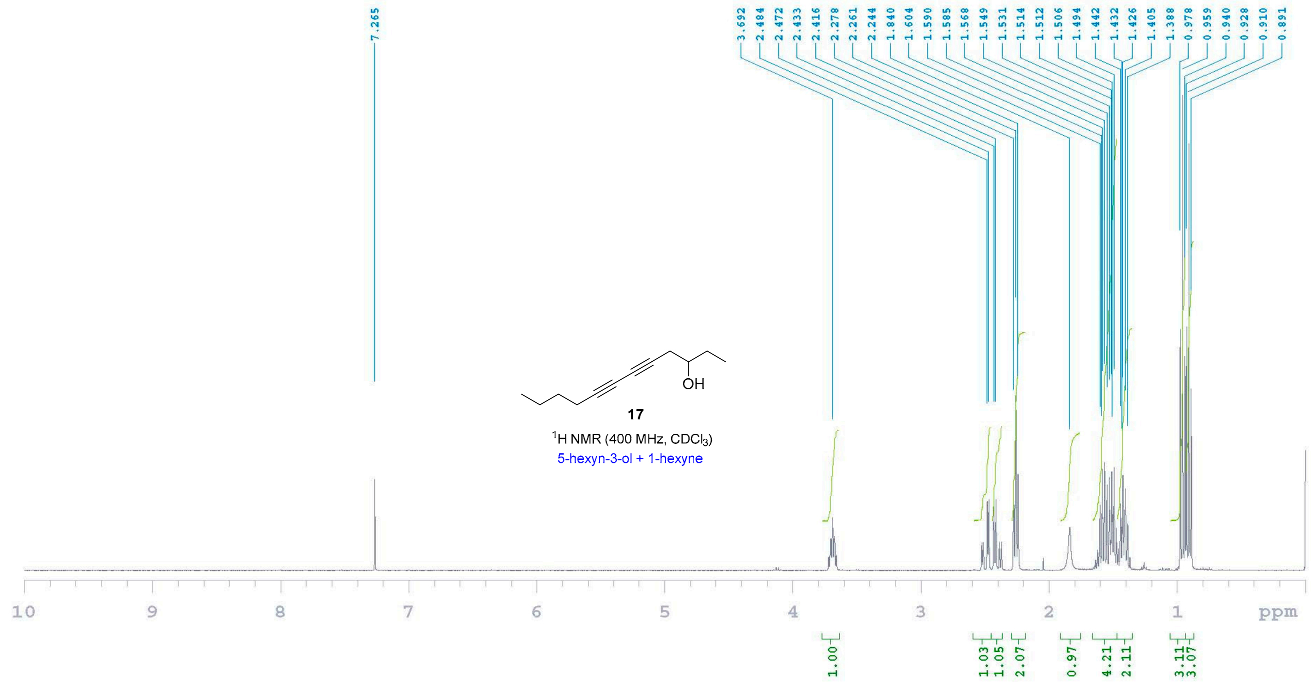
7.265
3.692 2.484 2.472 2.433 2.416 2.278 2.261 2.244 1.840 1.604 1.590 1.585 1.568 1.549 1.531 1.514 1.512 1.506 1.494 1.442 1.432 1.426 1.405 1.388 0.978 0.959 0.940 0.928 0.910 0.891
OH
17
^{1}H NMR (400 MHz, $CDCl_3$)
5-hexyn-3-ol + 1-hexyne
10 9 8 7 6 5 4 3 2 1 ppm
1.00 1.03 1.05 2.07 0.97 4.21 2.11 3.11 3.07

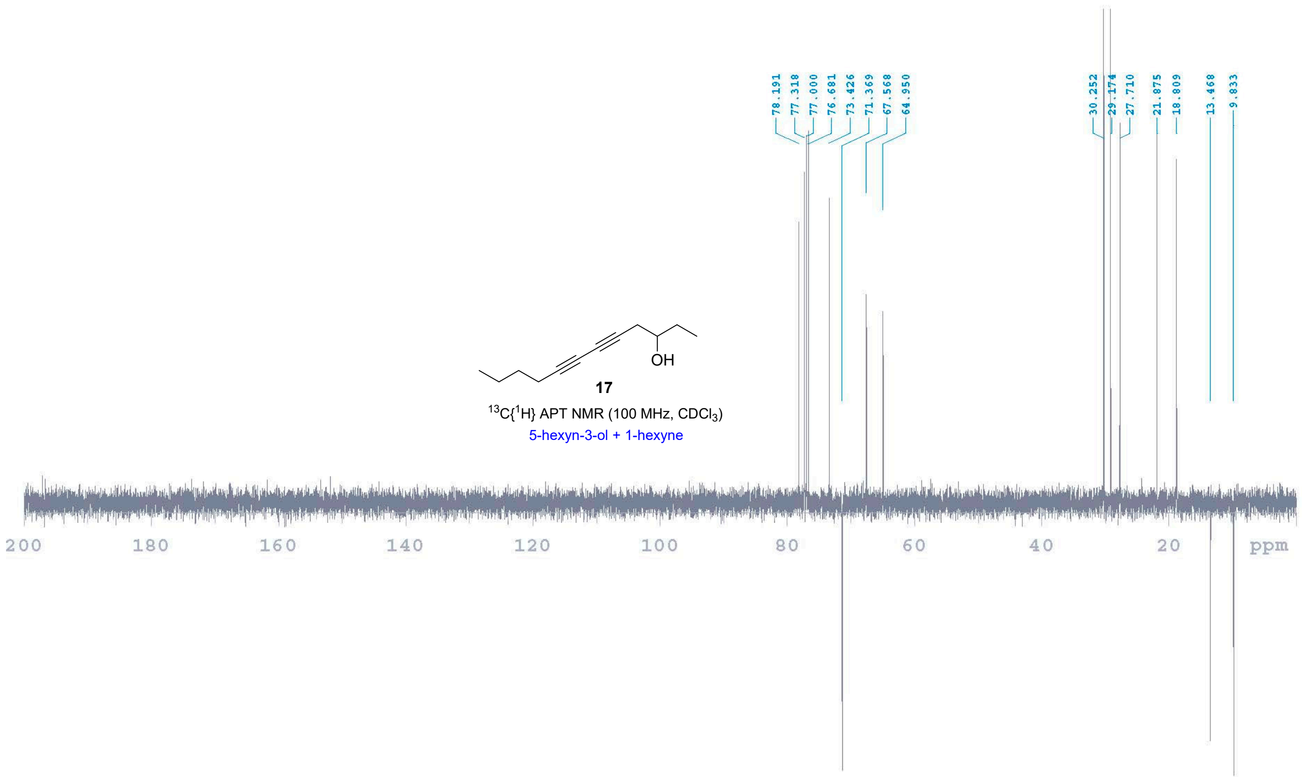
78.191 77.318 77.000 76.681 73.426 71.369 67.568 64.950
30.252 29.174 27.710 21.875 18.809 13.468 9.833
OH
17
^{13}C{^{1}H} APT NMR (100 MHz, $CDCl_3$)
5-hexyn-3-ol + 1-hexyne
200 180 160 140 120 100 80 60 40 20 ppm

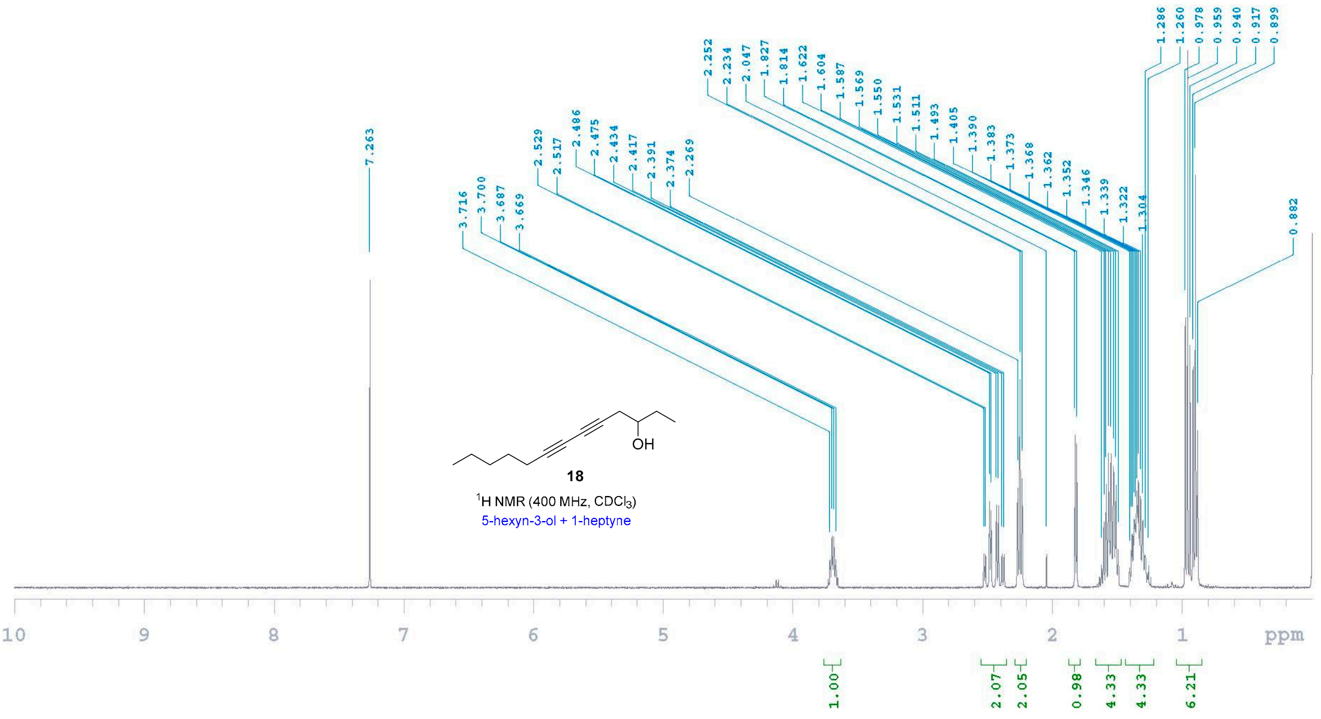
18
^{1}H NMR (400 MHz, CDCl$_3$)
5-hexyn-3-ol + 1-heptyne
OH
7.263
3.716
3.700
3.687
3.669
2.529
2.517
2.486
2.475
2.434
2.417
2.391
2.374
2.269
2.252
2.234
2.047
1.827
1.814
1.622
1.604
1.587
1.569
1.550
1.531
1.511
1.493
1.405
1.390
1.383
1.373
1.368
1.362
1.352
1.346
1.339
1.322
1.304
1.286
1.260
0.978
0.959
0.940
0.917
0.899
0.882
1.00
2.07
2.05
0.98
4.33
4.33
6.21
ppm

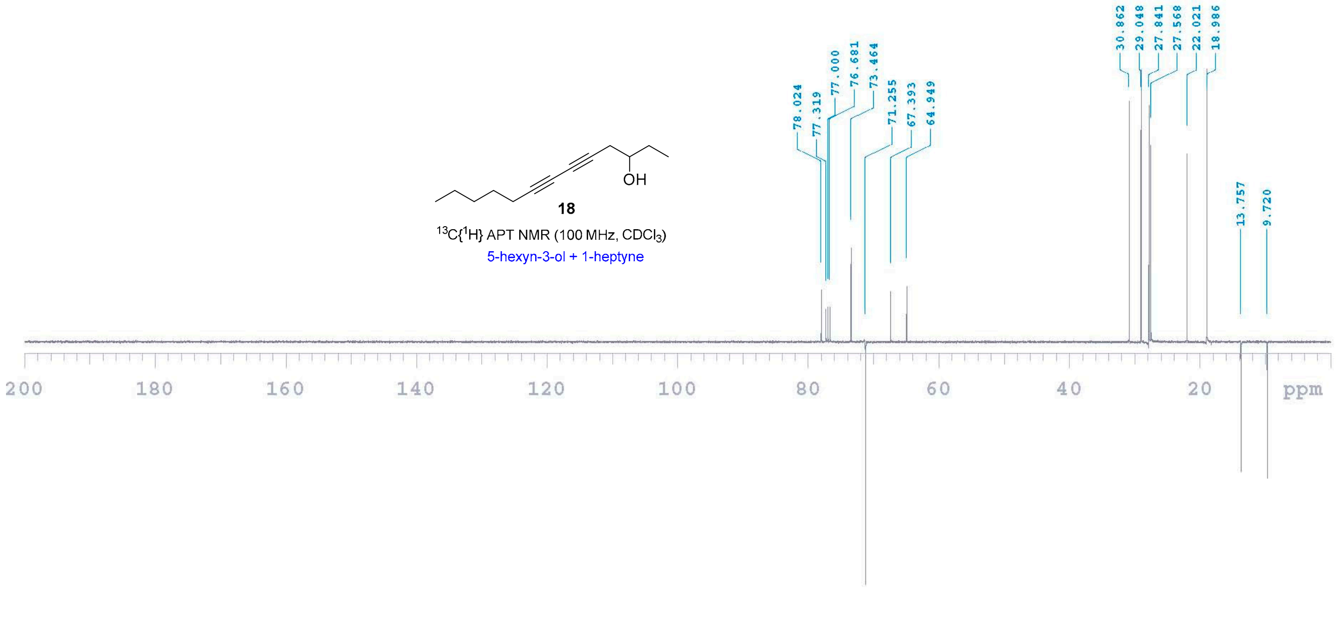
18
^{13}C{^{1}H} APT NMR (100 MHz, CDCl$_3$)
5-hexyn-3-ol + 1-heptyne
OH
78.024
77.319
77.000
76.681
73.464
71.255
67.393
64.949
30.862
29.048
27.841
27.568
22.021
18.986
13.757
9.720
ppm

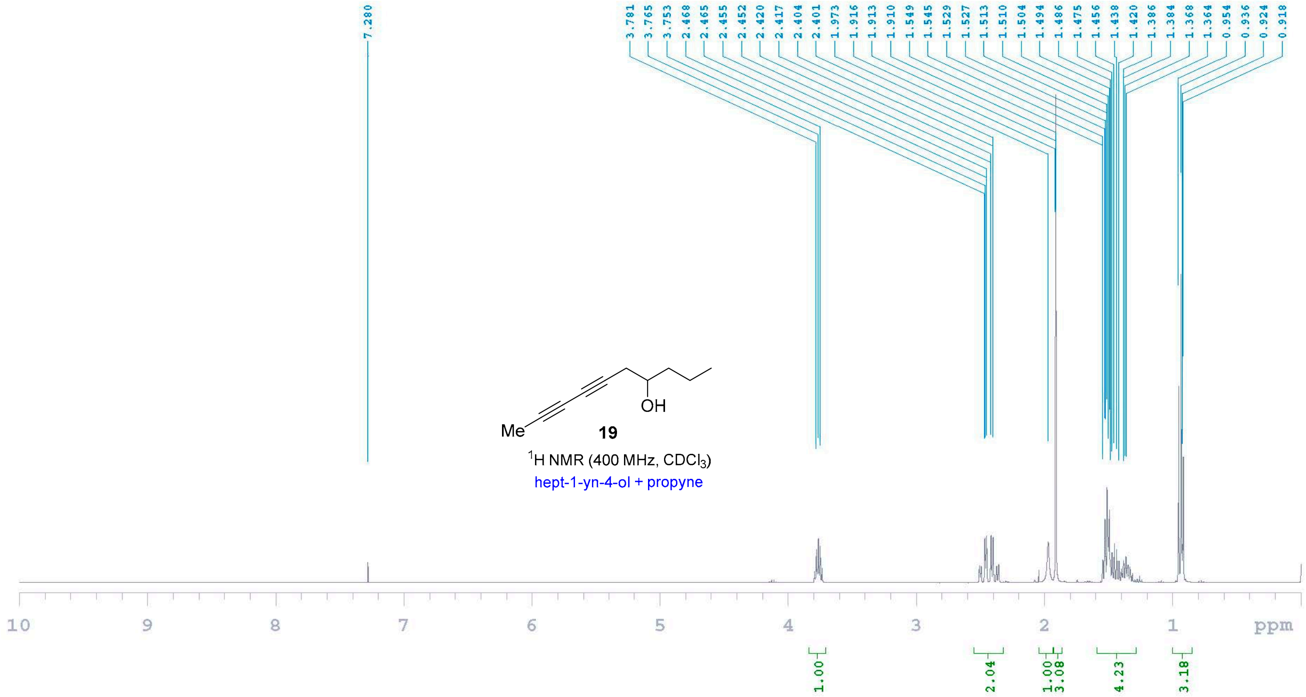
7.280
3.781 3.765 3.753 2.468 2.465 2.455 2.452 2.420 2.417 2.404 2.401 1.973 1.916 1.913 1.910 1.549 1.545 1.529 1.527 1.513 1.510 1.504 1.494 1.486 1.475 1.456 1.438 1.420 1.386 1.384 1.368 1.364 0.954 0.936 0.924 0.918
Me
OH
19
1H NMR (400 MHz, CDCl3)
hept-1-yn-4-ol + propyne
10 9 8 7 6 5 4 3 2 1 ppm
1.00 2.04 1.00 3.08 4.23 3.18

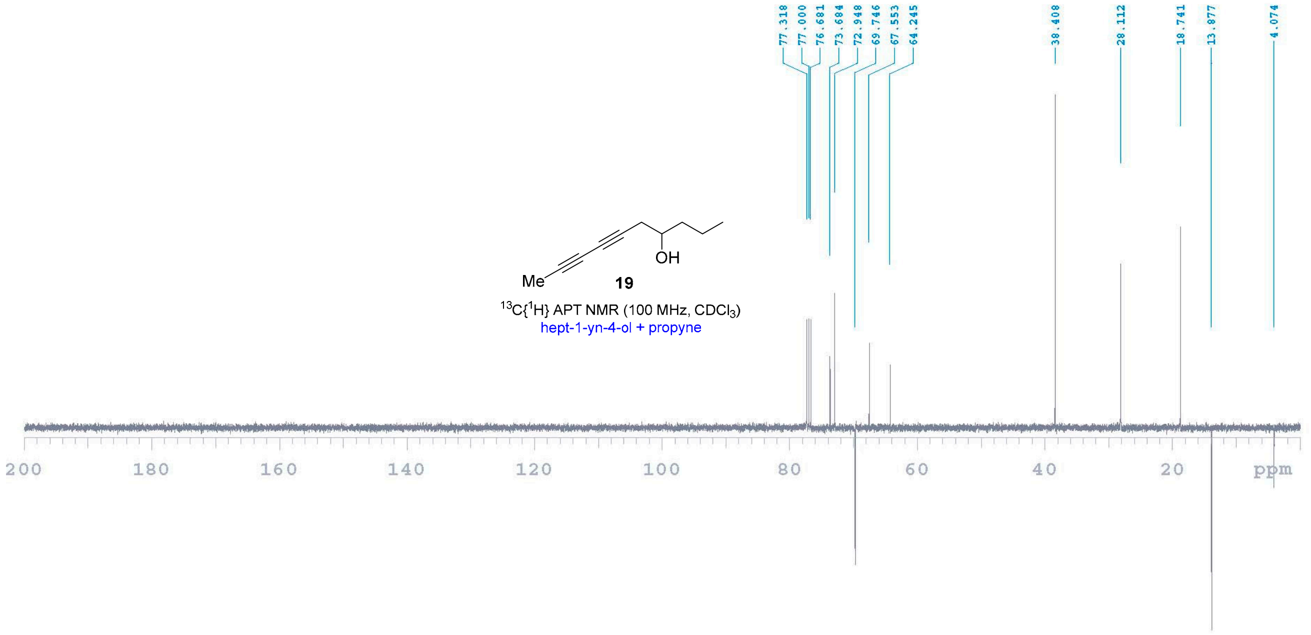
77.318 77.000 76.681 73.684 72.948 69.746 67.553 64.245 38.408 28.112 18.741 13.877 4.074
Me
OH
19
13C{1H} APT NMR (100 MHz, CDCl3)
hept-1-yn-4-ol + propyne
200 180 160 140 120 100 80 60 40 20 ppm

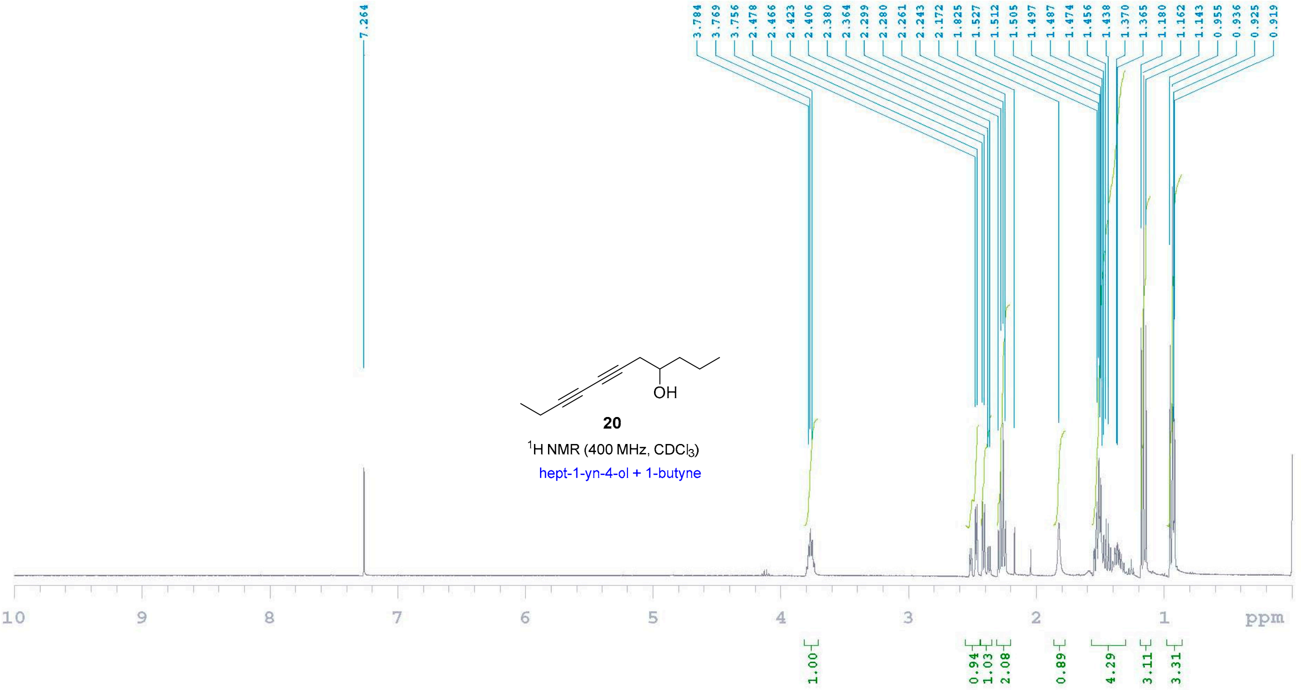
7.264
3.784
3.769
3.756
2.478
2.466
2.423
2.406
2.380
2.364
2.299
2.280
2.261
2.243
2.172
1.825
1.527
1.512
1.505
1.497
1.487
1.474
1.456
1.438
1.370
1.365
1.180
1.162
1.143
0.955
0.936
0.925
0.919
OH
20
1H NMR (400 MHz, CDCl3)
hept-1-yn-4-ol + 1-butyne
10
9
8
7
6
5
4
3
2
1
ppm
1.00
0.94
1.03
2.08
0.89
4.29
3.11
3.31

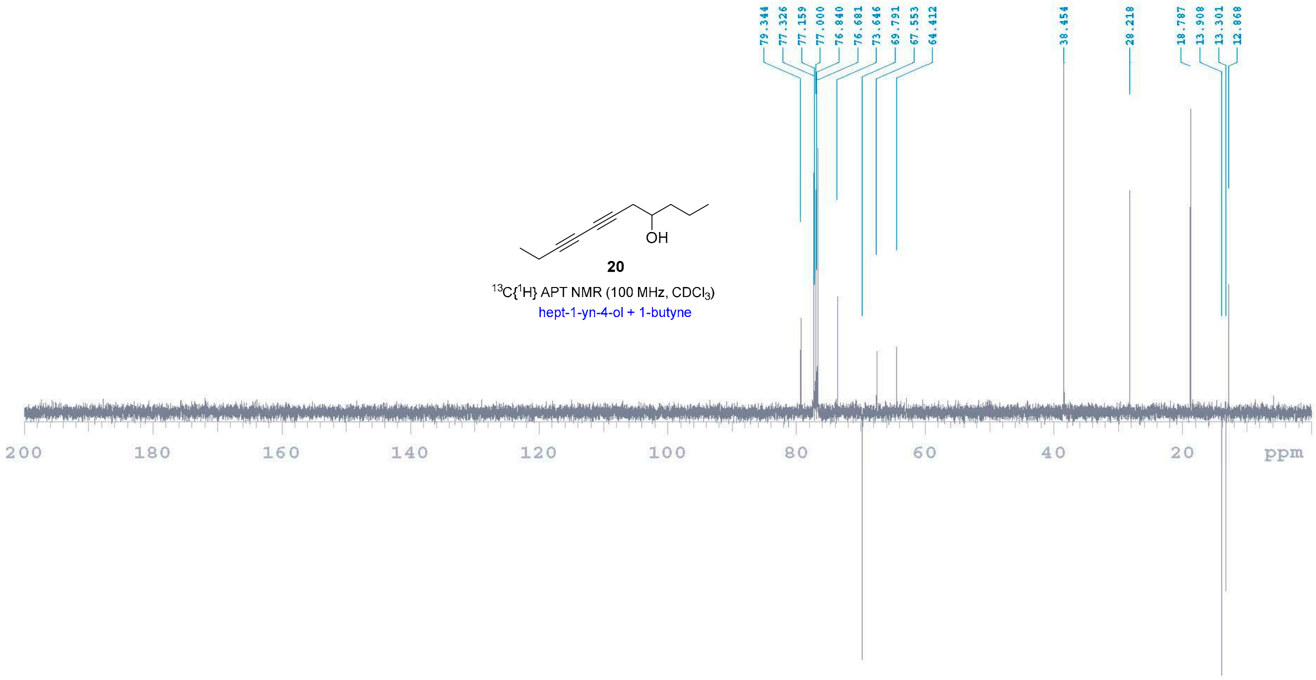
79.344
77.326
77.159
77.000
76.840
76.681
73.646
69.791
67.553
64.412
38.454
28.218
18.787
13.908
13.301
12.868
OH
20
13C{1H} APT NMR (100 MHz, CDCl3)
hept-1-yn-4-ol + 1-butyne
200
180
160
140
120
100
80
60
40
20
ppm

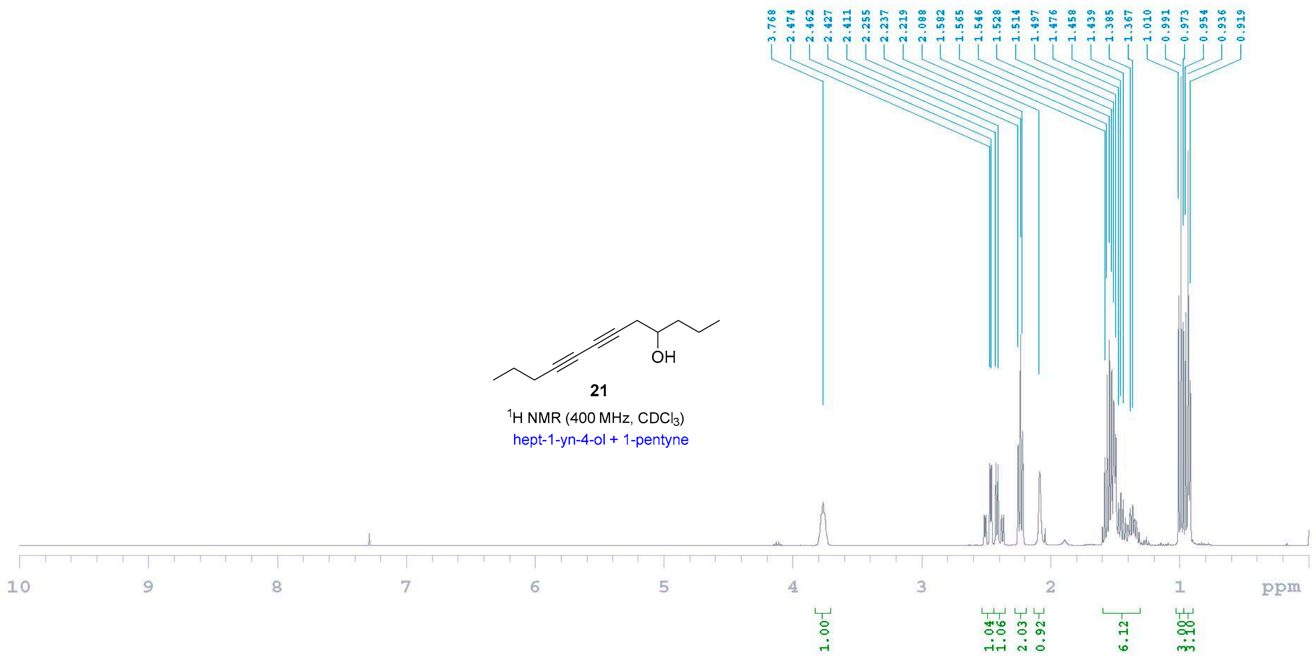
3.768 2.474 2.462 2.427 2.411 2.255 2.237 2.219 2.088 1.582 1.565 1.546 1.528 1.514 1.497 1.476 1.458 1.439 1.385 1.367 1.010 0.991 0.973 0.954 0.936 0.919
OH
21
^{1}H NMR (400 MHz, $CDCl_3$)
hept-1-yn-4-ol + 1-pentyne
10 9 8 7 6 5 4 3 2 1 ppm
1.00 1.04 1.06 2.03 0.92 6.12 3.00 3.10

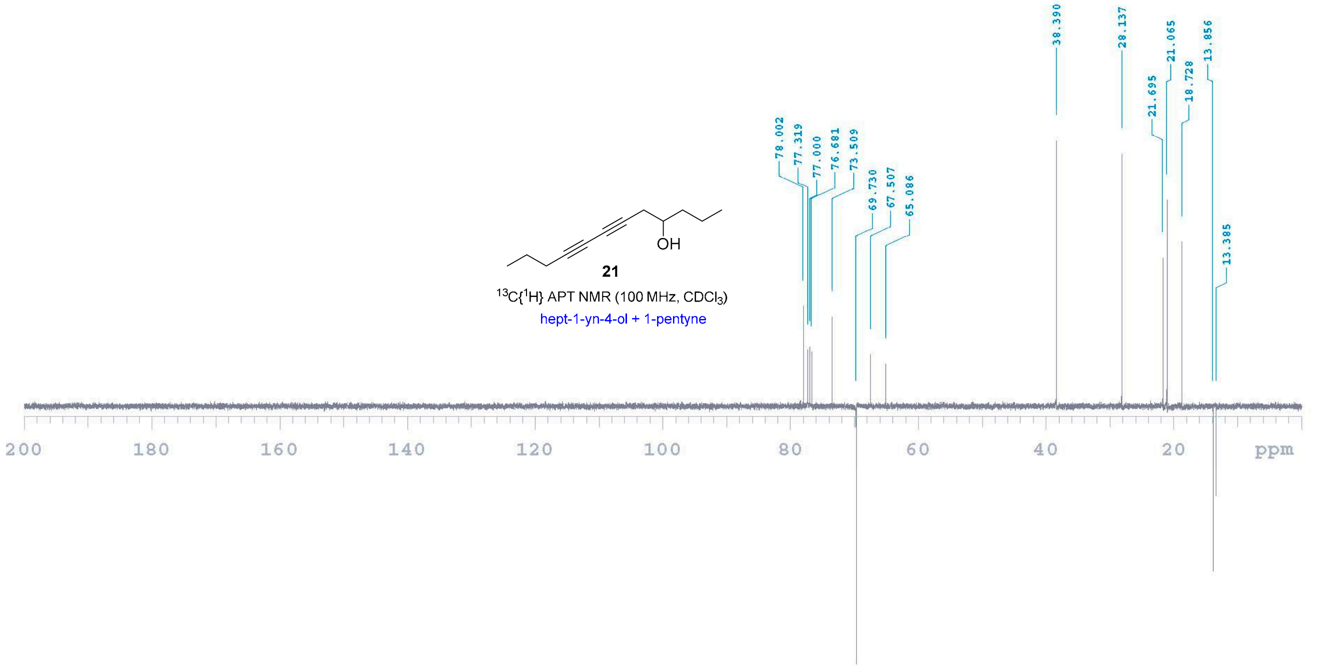
38.390 28.137 21.695 21.065 18.728 13.856
78.002 77.319 77.000 76.681 73.509 69.730 67.507 65.086 13.385
OH
21
^{13}C{^{1}H} APT NMR (100 MHz, $CDCl_3$)
hept-1-yn-4-ol + 1-pentyne
200 180 160 140 120 100 80 60 40 20 ppm

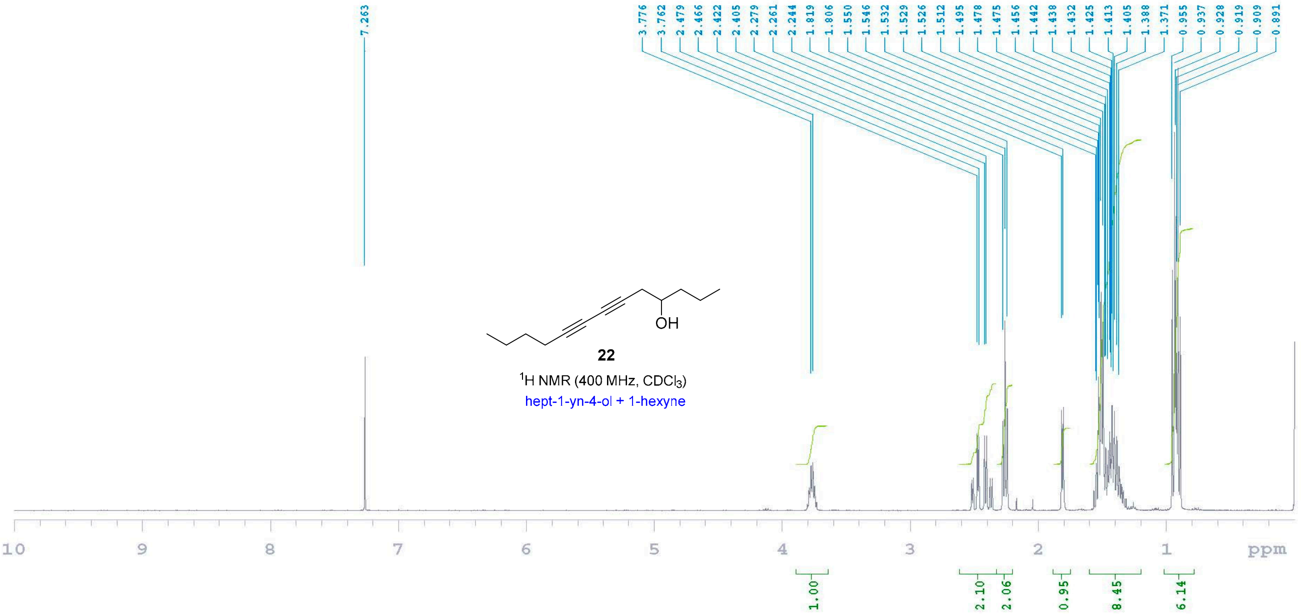
7.263
3.776 3.762 2.479 2.466 2.422 2.405 2.279 2.261 2.244 1.819 1.806 1.550 1.546 1.532 1.529 1.526 1.512 1.495 1.478 1.475 1.456 1.442 1.438 1.432 1.425 1.413 1.405 1.388 1.371 0.955 0.937 0.928 0.919 0.909 0.891
OH
22
1H NMR (400 MHz, CDCl3)
hept-1-yn-4-ol + 1-hexyne
10 9 8 7 6 5 4 3 2 1 ppm
1.00 2.10 2.06 0.95 8.45 6.14

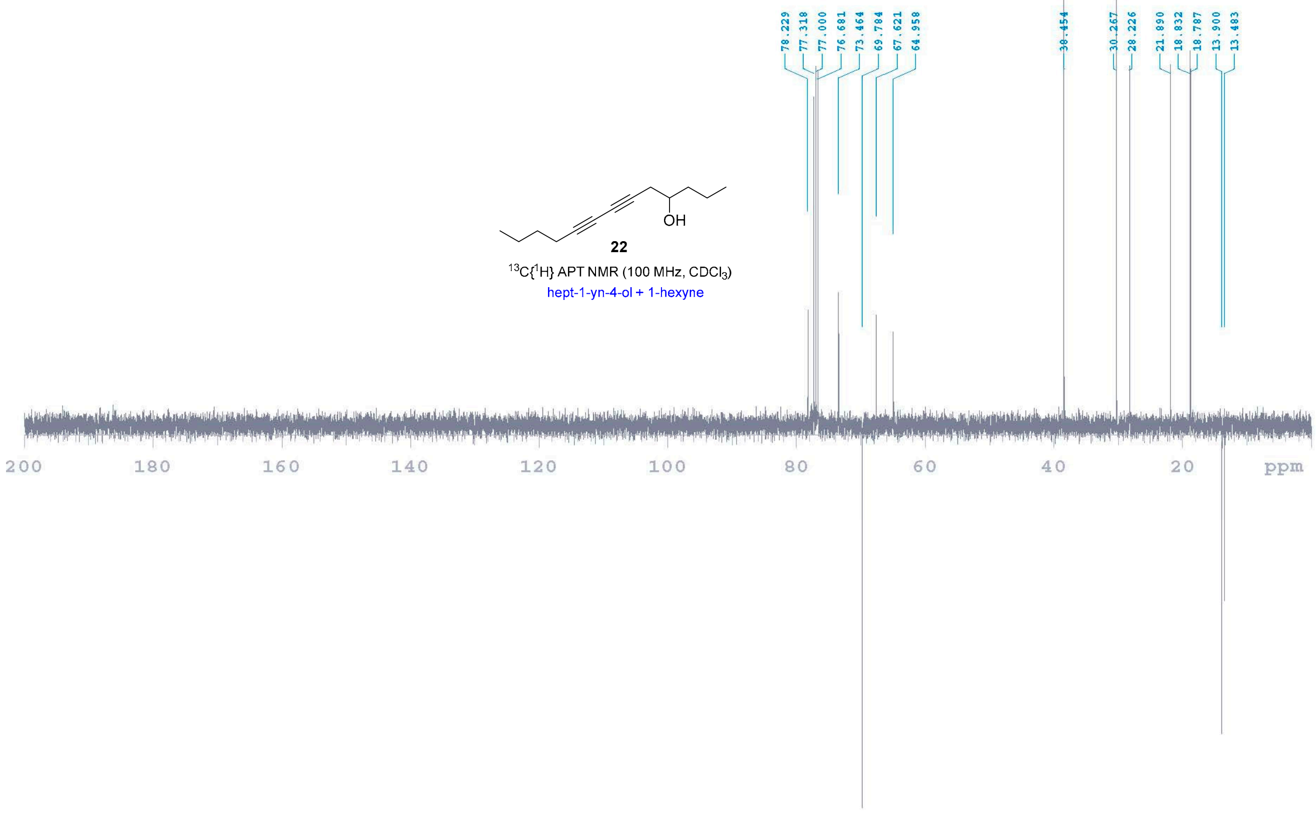
78.229 77.318 77.000 76.681 73.464 69.784 67.621 64.958 38.454 30.267 28.226 21.890 18.832 18.787 13.900 13.483
OH
22
13C{1H} APT NMR (100 MHz, CDCl3)
hept-1-yn-4-ol + 1-hexyne
200 180 160 140 120 100 80 60 40 20 ppm

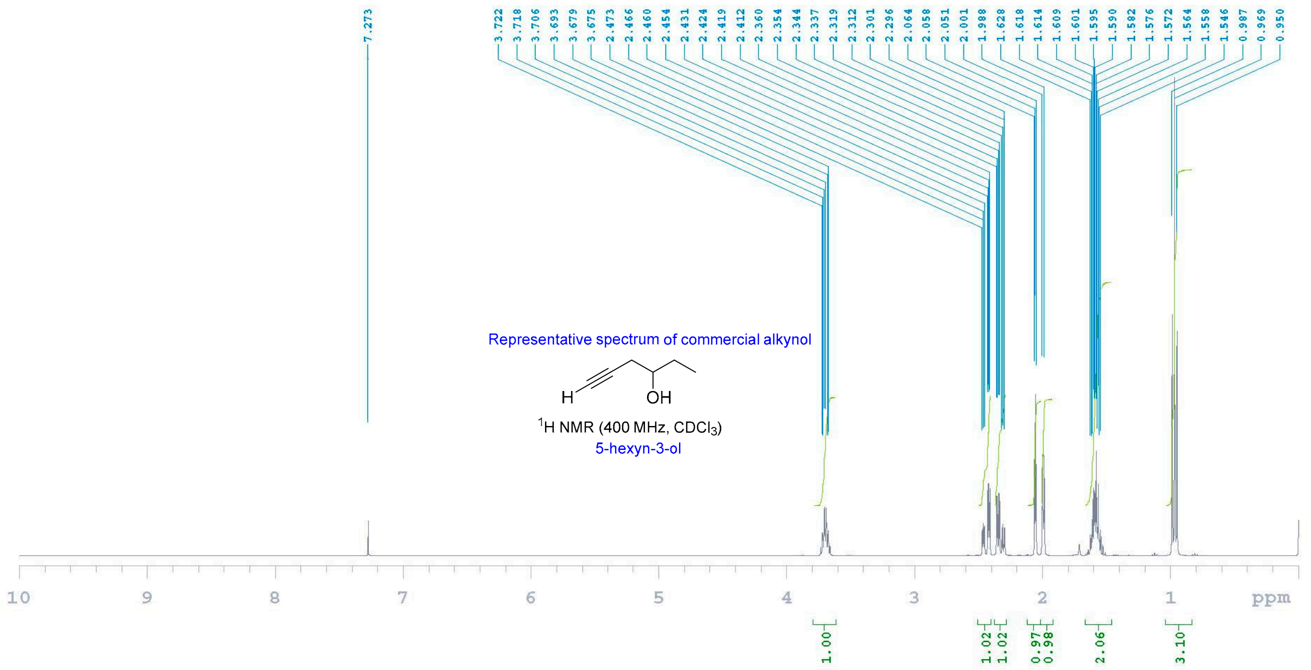

Representative spectrum of commercial alkynol
H
OH
1H NMR (400 MHz, CDCl3)
5-hexyn-3-ol
ppm

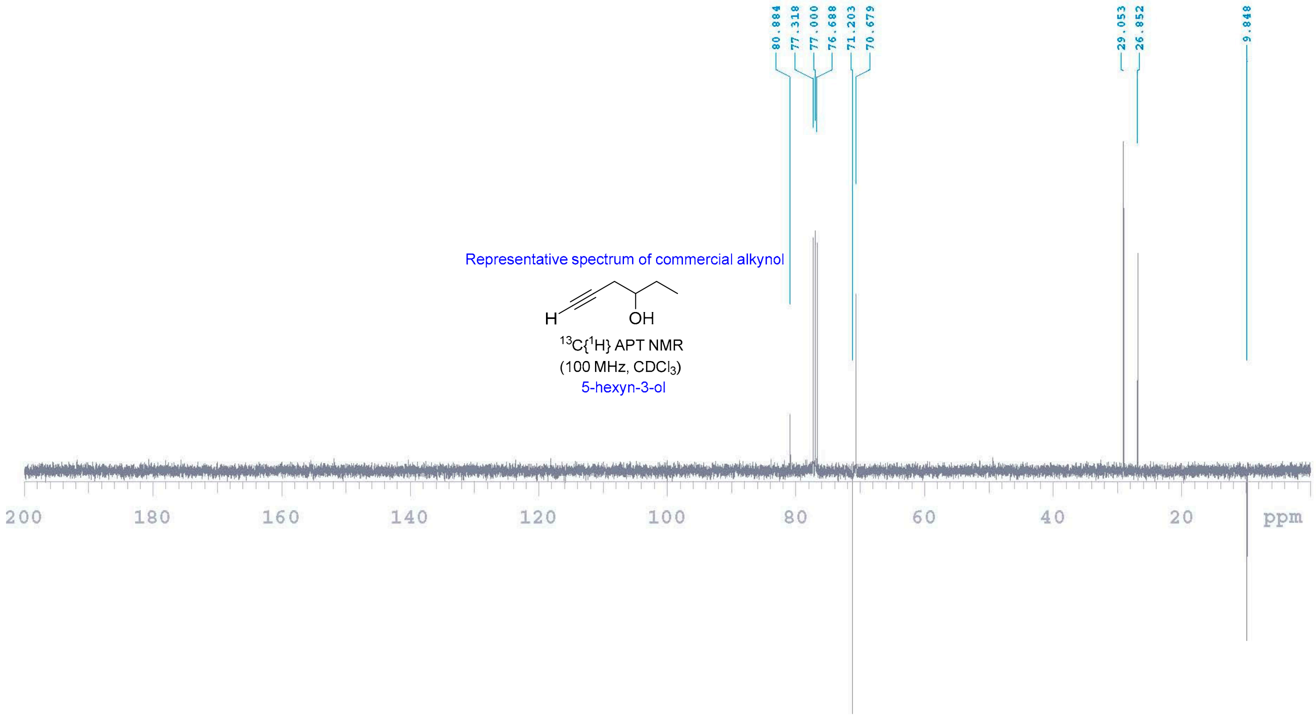

Representative spectrum of commercial alkynol
H
OH
13C{1H} APT NMR
(100 MHz, CDCl3)
5-hexyn-3-ol
80.884
77.318
77.000
76.688
71.203
70.679
29.053
26.852
9.848
ppm

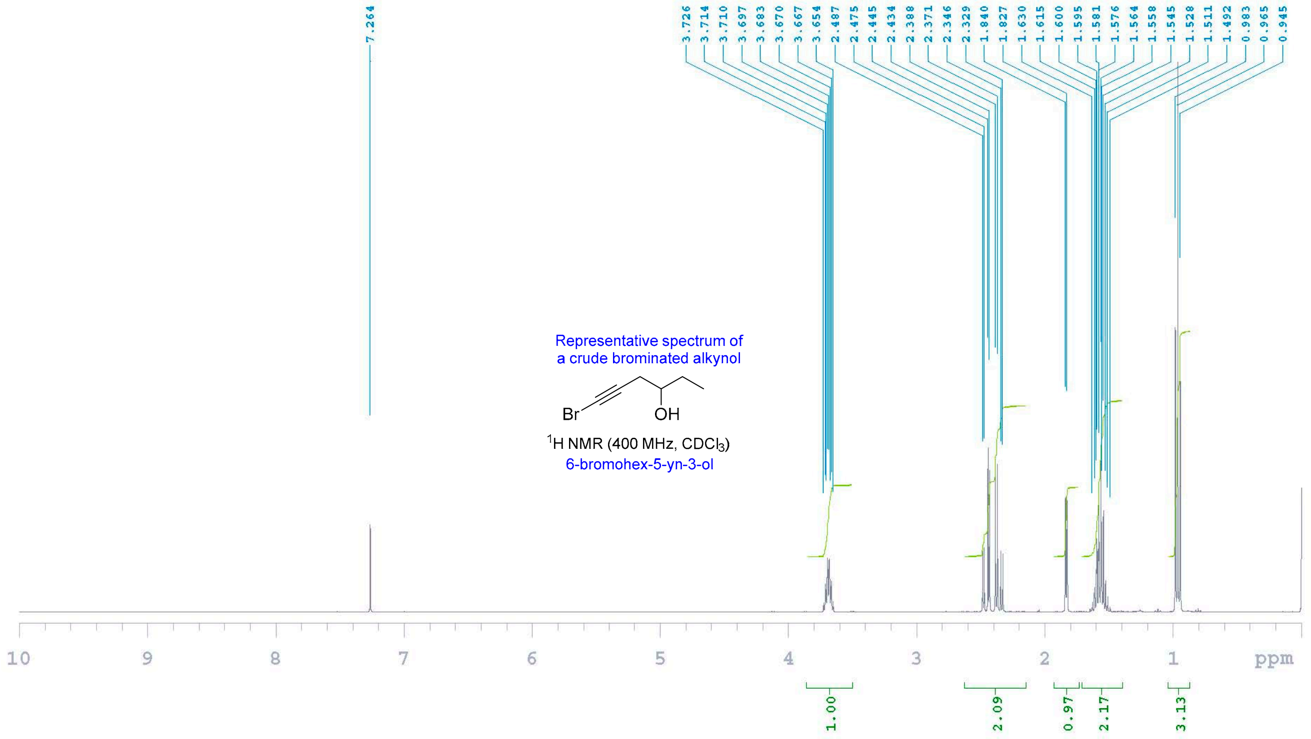
7.264
3.726
3.714
3.710
3.697
3.683
3.670
3.667
3.654
2.487
2.475
2.445
2.434
2.388
2.371
2.346
2.329
1.840
1.827
1.630
1.615
1.600
1.595
1.581
1.576
1.564
1.558
1.545
1.528
1.511
1.492
0.983
0.965
0.945
Representative spectrum of
a crude brominated alkynol
Br
OH
1H NMR (400 MHz, CDCl3)
6-bromohex-5-yn-3-ol
10
9
8
7
6
5
4
3
2
1
ppm
1.00
2.09
0.97
2.17
3.13

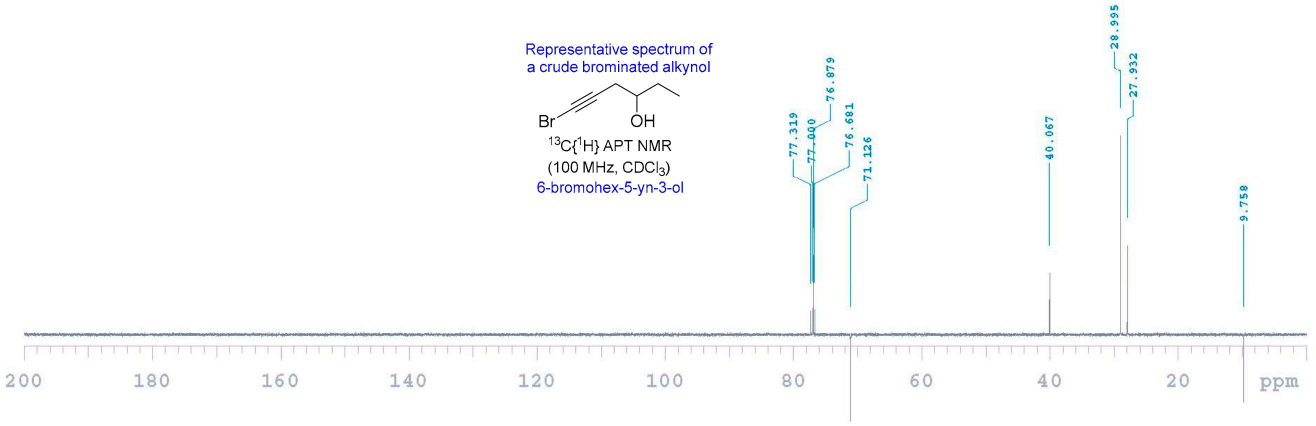
Representative spectrum of
a crude brominated alkynol
Br
OH
13C{1H} APT NMR
(100 MHz, CDCl3)
6-bromohex-5-yn-3-ol
77.319
77.000
76.879
76.681
71.126
40.067
28.995
27.932
9.758
200
180
160
140
120
100
80
60
40
20
ppm
